# Effects of heat shock on photosynthesis-related characteristics and lipid profile of C*ycas multipinnata* and *C. panzhihuaensis*

**DOI:** 10.1186/s12870-022-03825-0

**Published:** 2022-09-15

**Authors:** Huan Zhu, Yangyang Wu, Yanling Zheng

**Affiliations:** Key Laboratory of State Forestry and Grassland Administration for Biodiversity Conservation in Southwest China, Southwest Forestry University, Kunming, 650233 Yunnan China

**Keywords:** Chlorophyll fluorescence, Endangered plants, Lipidomics, Thermotolerance

## Abstract

**Background:**

*Cycas multipinnata* and *C. panzhihuaensis* are two attractive ornamental tree species. With the global climate change, the temperature in the natural habitats of both the species shows a marked rising trend. However, how the two species respond to extreme high temperatures are not clear.

Chlorophyll fluorescence parameters, chlorophyll content, chloroplast ultrastructure and lipid metabolism in the two species were determined following plant exposure to heat stress.

**Results:**

The results demonstrated that the photosynthetic efficiency decreased significantly in both the species following heat shock and recovery, but to a greater extent in *C. panzhihuaensis*. Compared to the control, chlorophyll content of *C. multipinnata* did not change significantly following heat stress and recovery. However, chlorophyll content of *C. panzhihuaensis* increased significantly after 1 d of recovery in comparison with the control. Chloroplast ultrastructures of *C. panzhihuaensis* were more severely affected by heat shock than *C. multipinnata*. *C. multipinnata* and *C. panzhihuaensis* followed a similar change trend in the amounts of most of the lipid categories after heat stress. However, only the amounts of lysophospholipids and fatty acyls differed significantly between the two species following heat treatment. Additionally, the unsaturation levels of the major lipid classes in *C. multipinnata* were significantly lower than or equal to those in *C. panzhihuaensis*.

**Conclusions:**

*C. multipinnata* was less affected by extremely high temperatures than *C. panzhihuaensis.* The differential stability of chlorophyll and chloroplast ultrastructure and the differential adjustment of lipid metabolism might contribute to the different responses to heat shock between the two species.

**Supplementary Information:**

The online version contains supplementary material available at 10.1186/s12870-022-03825-0.

## Background

With the change of global climate, the magnitude and frequency of extreme high temperatures have increased in the past several decades and the global mean temperatures are likely to increase by 3.7 ± 1.1 °C at the end of twenty-first century [1, 2]. In many parts of the world, seasonal warming variation may result in the hottest temperatures rising more than the annual mean [3, 4]. Being sessile, plants are adversely impacted by heat stress in terms of growth, reproduction, and yield [5, 6]. Among the physiological processes of plants, photosynthesis is of vital importance but extremely sensitive to high temperatures [7, 8]. High temperatures can reduce the capacity for photochemical utilization of absorbed light energy due to the degradation of chlorophyll, disruption of the chloroplast ultrastructure and inactivation of ribulose bisphosphate carboxylase/oxygenase [9–11]. Therefore, photoinhibition which is caused by excess light energy can be easily induced by high temperatures [12]. Photosystem II (PSII) plays key roles in the light reactions of photosynthesis. However, PSII is considered as the most heat-sensitive component of the photosynthetic apparatus [8, 13]. As chlorophyll fluorescence can indicate the light absorption, transmission, distribution and energy dissipation of photosystem II, it has been widely used to obtain information on plant photosynthetic performance [14, 15].

The membrane is an indispensable platform for plant growth, signaling, and development [16]. However, the membrane is particularly susceptible to injury from adverse conditions, including extreme temperatures [17, 18]. For example, chloroplast and thylakoid membranes are easily damaged by heat stress [13, 18]. As the crucial component of membranes, specific lipid composition determines the identity and function of a specific compartment [16, 19]. Additionally, lipids are involved in signaling transduction and energy storage, which play essential roles in plant development and adaptation to adverse growth conditions [20–22]. However, lipid peroxidation and some changes in lipid composition can result in membrane injury, electrolyte leakage, metabolic dysfunction, and even the ultimate death of plant cells [18, 23]. It has been reported that lipids play pivotal roles in heat stress management of plants [24, 25]. To try to adjust to increasing ambient temperatures, some plants could regulate the lipid composition to adjust membrane fluidity and maintain membrane integrity [24, 26]. The decrease in lipid unsaturation levels and increase in the proportions of bilayer-forming lipids enable some plants to maintain membrane stability as the temperature increases [24, 27, 28].

Species of *Cycas* are attractive ornamental trees and there are more than 20 *Cycas* species distributed in the subtropical and tropical areas of China [29, 30]. However, owing to overexploitation for horticultural trade, habitat degradation, and some other factors, most species have few populations and small population size, with limited distribution [31, 32]. Among these species, *C. panzhihuaensis* and *C. multipinnata* possess different morphological characteristics and geographic distribution. *C. panzhihuaensis* is endemic to the dry-hot valleys of the Jinsha River in southwest China which experiences south-subtropical semi-arid valley climate [33, 34]. The species is mainly distributed at an altitude of 1100–2000 m. *C. multipinnata* is a plant species with extremely small populations and endemic to a limited area in the Red River gorge in the southeastern Yunnan province in southwest China, with a disjunct occurrence in northern Vietnam. This species is mainly distributed in tropical limestone hill seasonal rainforests or montane rainforests at an altitude of 150–1100 m [29, 35]. Although *C. panzhihuaensis* and *C. multipinnata* distribute in different locations and habitats, the extreme air temperatures of their habitats can exceed 40 °C [29, 33–35], and the soil surface temperatures are predicted to be much more higher [36]. Climate is a primary control on species distributions and the climate change including the occurrence of extreme high temperatures can increase the extinction risk of species with narrow geographic or climatic distributions [37]. Compared to the higher latitude species, tropical species are more at risk from climate warming as further warming may bring these species closer to their upper thermal limits [38, 39]. Therefore, extreme high temperature events might put these species in a more dangerous situation of extinction. Plant thermotolerance depends on many factors, including the habitat conditions and plant genetic basis [40, 41]. *C. multipinnata* is distributed at lower latitude and altitude areas than *C. panzhihuaensis*. We hypothesized that *C. multipinnata* might be more tolerant to heat stress than *C. panzhihuaensis*. However, some species that naturally distribute in warmer and drier conditions are less heat tolerant than species that occur in cooler and moister sites [42]. How the two species respond to heat stress and whether they differ in thermotolerance have not been understood.

The effects of high temperature on plants depend on the intensity and exposure time [43]. Heat shock treatment is often used to evaluate the plant thermotolerance and related mechanisms. Depending on species, the chosen temperatures of heat shock are generally extremely high ranging from about 40 °C to more than 50 °C and the exposure duration is generally short ranging from several minutes to several hours [12, 44, 45]. To understand the performance of both the species under the forecasted climate warming, plants of *C. multipinnata* and *C. panzhihuaensis* were subjected to heat shock to determine 1) the thermotolerance of *C. multipinnata* and *C. panzhihuaensis* by measuring chlorophyll content, chloroplast ultrastructure and chlorophyll fluorescence parameters; 2) the detailed lipid profiles to explore possible lipid signatures related to thermotolerance in these species. The results can provide theoretical basis for the introduction, acclimatization and cultivation of *C. multipinnata* and *C. panzhihuaensis*.

## Results

### Chlorophyll fluorescence parameters and chlorophyll content following heat shock and recovery

Species and treatment had significant effects on Fv/Fm and significant interactive effects were found between the two factors. Compared to the controls, Fv/Fm decreased significantly following heat stress in both *C. multipinnata* and *C. panzhihuaensis* (Table 1). After 1 d of recovery, Fv/Fm did not change in *C. multipinnata* but further decreased significantly in *C. panzhihuaensis* in comparison with those of the heat-treated plants. Y(II), qP, rETR and Y(NO) were significantly affected by treatment and species, with significant interaction between the two factors. Heat induced a significant decrease of Y(II), qP, and rETR and a significant increase of Y(NO) for both the species, however, to a lesser extent in *C. multipinnata*. Y(NPQ) was only significantly affected by treatment, with no significant interaction between treatment and species. Compared to the control, Y(NPQ) decreased significantly in both *C. panzhihuaensis* and *C. multipinnata* immediately following heat stress. After 1 d of recovery, Y(NPQ) remained unchanged in *C. multipinnata* but further decreased significantly in *C. panzhihuaensis* in comparison with those of heat-treated plants. qN was only impacted by species, with significant interaction between treatment and species. Compared to the control, qN decreased significantly in *C. multipinnata* but did not change significantly in *C. panzhihuaensis* following heat stress and 1 d of recovery. After 1 d of recovery, Fv/Fm, Y(II), qP and rETR of *C. multipinnata* were all significantly higher, but both Y(NO) and qN were significantly lower, in comparison with those of *C. panzhihuaensis* (Table 1). Taken together, these results showed that photosynthesis of *C. panzhihuaensis* was more sensitive to heat stress than that of *C. multipinnata*.

**Table 1:** The chlorophyll fluorescence following high shock (H) and recovery (R) in *C. multipinnata* and *C. panzhihuaensis*

	Fv/Fm	Y(II)	Y(NPQ)	Y(NO)	qp	qN	rETR
*C. multipinnata*							
Control	0.819 ± 0.017a	0.460 ± 0.069a	0.292 ± 0.100a	0.248 ± 0.034b	0.792 ± 0.021a*	0.651 ± 0.151a	116.8 ± 17.570a
H	0.322 ± 0.037b	0.120 ± 0.019b*	0.180 ± 0.063b	0.700 ± 0.074a*	0.438 ± 0.054c*	0.473 ± 0.107b	30.400 ± 4.561b*
R	0.357 ± 0.030b*	0.147 ± 0.010b*	0.114 ± 0.017b	0.739 ± 0.018a*	0.547 ± 0.031b*	0.363 ± 0.035b*	36.200 ± 2.490b*
*C. panzhihuaensis*							
Control	0.833 ± 0.003a	0.465 ± 0.050a	0.260 ± 0.046a	0.275 ± 0.016c	0.561 ± 0.050a	0.635 ± 0.048ab	117.4 ± 12.740a
H	0.339 ± 0.034b	0 ± 0b	0.178 ± 0.024b	0.822 ± 0.024b	0 ± 0b	0.583 ± 0.061b	0 ± 0b
R	0.160 ± 0.032c	0 ± 0b	0.085 ± 0.037c	0.915 ± 0.037a	0 ± 0b	0.791 ± 0.191a	0 ± 0b
Significance							
Treatment	*	*	*	*	*	ns	*
Species	*	*	ns	*	*	*	*
Treatment × species	*	*	ns	*	*	*	*

Two-way ANOVA analysis was performed in the general linear model

*rETR* relative electron transport rate, *Fv/Fm* the maximum quantum yield of photosystem II (PSII); *qN* non-photochemical quenching coefficient, *qP* photochemical quenching coefficient, *Y(II)* effective quantum yield of PS II, *Y(NO)* non-regulated non-photochemical energy loss in PS II, *Y(NPQ)* regulated non-photochemical energy loss in PS II

^*^ indicates *P* ≤ 0.05 and ns indicates not significant. For the same species, different letters in the same column are significantly different between treatments according to One-way ANOVA at *P* ≤ 0.05. For the same treatment, * is significantly different between species according to independent samples T-test at *P* ≤ 0.05. Values shown are the mean ± SD, *n* = 5

**Fig. 1:**
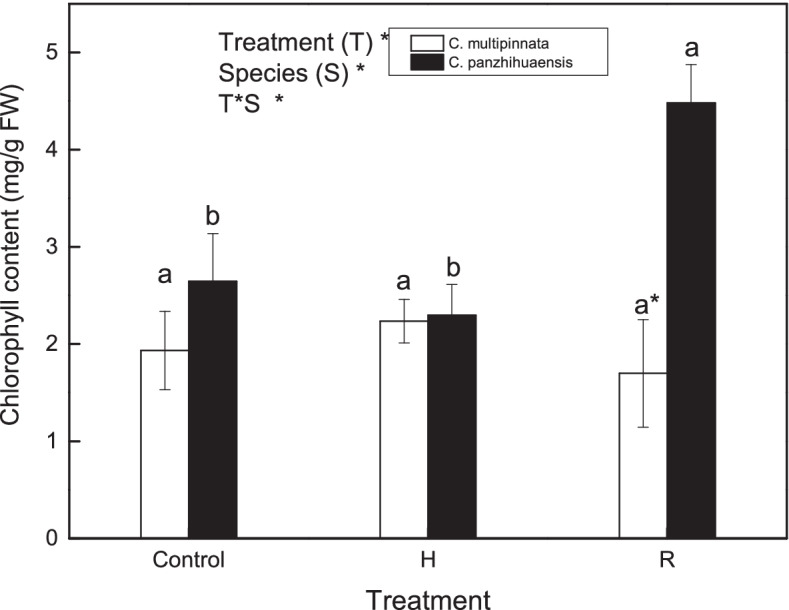
The content of chlorophyll following high shock (H) and recovery (R) in *C. multipinnata* and *C. panzhihuaensis*. Two-way ANOVA analysis was performed in the general linear model. * indicates *P* ≤ 0.05 and ns indicates not significant. For the same species, different letters in the same column are significantly different between treatments according to One-way ANOVA at *P* ≤ 0.05. For the same treatment, * is significantly different between species according to independent samples T-test at *P* ≤ 0.05. Values shown are the mean ± SD, *n* = 5

Chlorophyll content was significantly affected by treatment and species, with significant interaction between the two factors (Fig. 1). Chlorophyll content of *C. multipinnata* and *C. panzhihuaensis* did not change significantly immediately following heat shock (Fig. 1). After 1 d of recovery, the chlorophyll content remained unchanged in *C. multipinnata* but increased significantly in *C. panzhihuaensis*. Based on our observations, some leaves of *C. multipinnata* bleached only at the leaf tip but most leaves of *C. panzhihuaensis* turned brown from leaf tip to base after 7 d of recovery (Additional file 1). The results showed that the chlorophyll degradation of the two species occurred gradually to different extents during the recovery from heat stress.

#### Effects of heat shock on the chloroplast ultrastructure

**Fig. 2:**
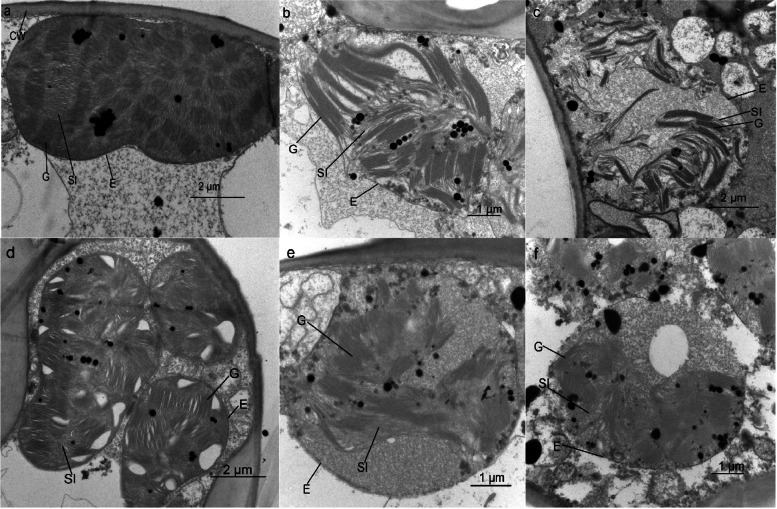
The chloroplast ultrastructure of mesophyll cells in *C. multipinnata* and *C. panzhihuaensis* immediately following heat stress. **a**, **b** and **c** show the normal, mildly affected and severely affected chloroplast ultrastructures of *C. multipinnata*, respectively; **d**, **e** and **f** show the normal, mildly affected and severely affected chloroplast ultrastructures of *C. panzhihuaensis*, respectively. CW: cell wall; E; envelope of chloroplast; G: granum; Sl: stroma lamellae

In the control, the chloroplasts of *C. multipinnata* and *C. panzhihuaensis* were intact, with an orderly arrangement of grana and stroma lamellae (Fig. 2a, d). Following heat stress, the arrangements of grana and stroma lamellae were little affected (Fig. 2b) or were disordered (Fig. 2c) in *C. multipinnata*. Compared to *C. multipinnata*, the chloroplasts of *C. panzhihuaensis* were more adversely affected by heat shock. Even though the envelop can remain intact, the arrangements of grana and stroma lamellae were disordered (Fig. 2e). Some chloroplasts were characterized by a disintegrating envelope and irregularly arranged grana and stroma lamellae (Fig. 2f). Based on these results, the chloroplast ultrastructure of *C. panzhihuaensis* was more severely affected by heat stress than that of *C. multipinnata*.

#### Effects of heat shock on the amount of each lipid category

**Fig. 3:**
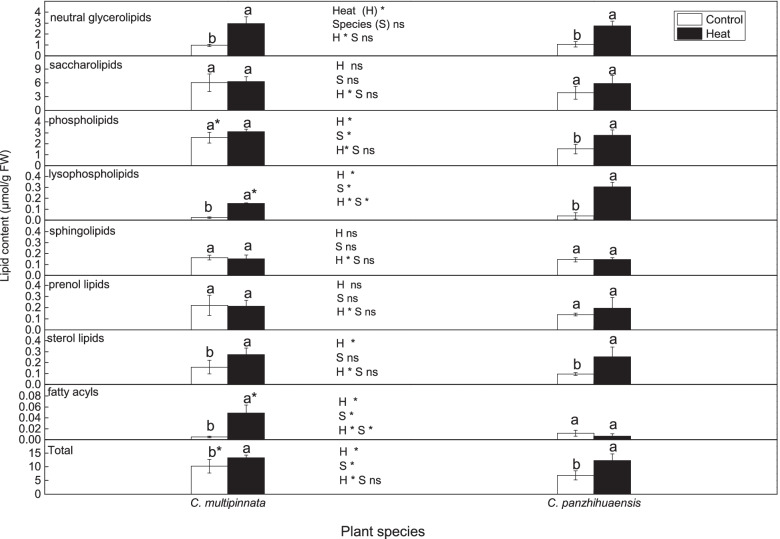
The content of each lipid category following heat stress in *C. multipinnata* and *C. panzhihuaensis*. Two-way ANOVA analysis was performed in the general linear model. * indicates *P* ≤ 0.05 and ns indicates not significant. For the same species, different letters in the same column are significantly different between treatments according to independent samples T-test at *P* ≤ 0.05. For the same treatment, * is significantly different between species at *P* ≤ 0.05. Values shown are the mean ± SD, *n* = 5

Eight main lipid categories (phospholipids, saccharolipids, neutral glycerolipids, lysophospholipids, sphingolipids, prenol lipids, sterol lipids, fatty acyls) including 26 lipid classes and 613 lipid species were determined (Additional file 2). The two species followed a similar change trend in the amounts of most lipid categories after heat stress (Fig. 3). Saccharolipids, sphingolipids, and prenol lipids were not significantly affected by heat and species. Neutral glycerolipids and sterol lipids were only significantly affected by heat stress. The amounts of neutral glycerolipids and sterol lipids increased markedly following heat stress, by 206.01% and 71.50% respectively in *C. multipinnta*, and by 160.46% and 165.31% respectively in *C. panzhihuaensis*. Heat and species had significant effects on lysophospholipids and fatty acyls with significant interaction between the two factors. Compared to the controls, the amounts of lysophospholipids increased by 590.69% and 692.87% in *C. multipinnata* and *C. panzhihuaensis*, respectively, following heat stress. Compared to the controls, the amount of fatty acyls did not change significantly in *C. panzhihuaensis* but increased by 864.54% in *C. multipinnata* following heat shock. Phospholipids and total lipids were significantly affected by heat and species. Phospholipids remained unchanged in *C. multipinnata* but increased significantly by 81.32% in *C. panzhihuaensis* after heat shock. Heat stress induced the accumulation of the total lipids in *C. multipinnata* and *C. panzhihuaensis*, by 29.56% and 78.83%, respectively. The amounts of most lipid categories did not differ significantly between the two species after heat stress. However, compared to those in *C. panzhihuaensis*, the amount of lysophospholipids was markedly lower and that of fatty acyls was significantly higher in *C. multipinnata* following heat stress. These results showed that most of the lipid categories presented a similar change trend and non-significant difference in ultimate amount between *C. multipinnata* and *C. panzhihuaensis* after heat shock.

#### Effects of heat stress on the amounts of each lipid class of the glycerolipids

**Fig. 4:**
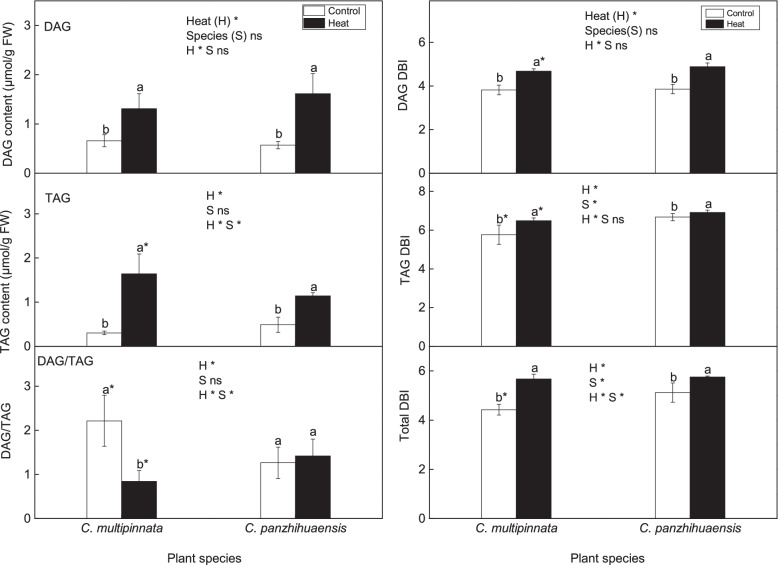
The content and DBI (double bond index) of diacylglycerol (DAG) and triacylglycerol (TAG) following heat stress in *C. multipinnata* and *C. panzhihuaensis.* Two-way ANOVA analysis was performed in the general linear model. * indicates *P* ≤ 0.05 and ns indicates not significant. For the same species, different letters in the same column are significantly different between treatments according to independent samples T-test at *P* ≤ 0.05. For the same treatment, * is significantly different between species at *P* ≤ 0.05. Values shown are the mean ± SD, *n* = 5

Both diacylglycerol (DAG) and triacylglycerol (TAG) amounts were significantly affected by heat stress (Fig. 4). Moreover, heat and species had interactive effects on TAG content. The amounts of DAG and TAG improved significantly following heat stress, by 98.35% and 438.87% respectively, in *C. multipinnata*, and by 183.71% and 133.43% respectively, in *C. panzhihuaensis* (Fig. 4). Heat had significant effects on the ratio of DAG to TAG (DAG/TAG), with significant interaction between heat and species. DAG/TAG declined significantly in *C. multipinnata* but remained unchanged in *C. panzhihuaensis* following heat shock (Fig. 4). In comparison with that of *C. panzhihuaensis*, the DAG/TAG of *C. multipinnata* was significantly higher in non-stressed plants but significantly lower in heat-stressed plants. The results suggested that heat induced the accumulation of DAG and TAG in both the species, but to a different extent.

**Fig. 5:**
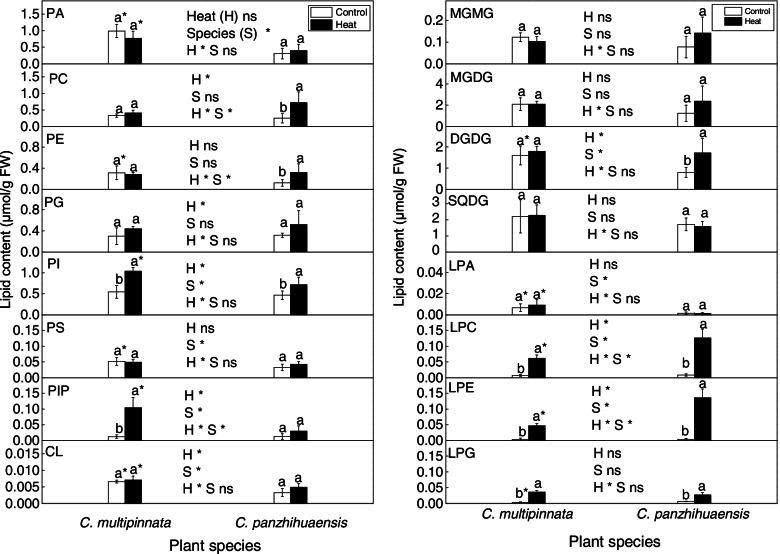
The amount of each lipid class of phospholipids, saccharolipids and lysophospholipids following heat stress in *C. multipinnata* and *C. panzhihuaensis*. Two-way ANOVA analysis was performed in the general linear model. * indicates *P* ≤ 0.05 and ns indicates not significant. For the same species, different letters in the same column are significantly different between treatments according to independent samples T-test at *P* ≤ 0.05. For the same treatment, * is significantly different between species at *P* ≤ 0.05. Values shown are the mean ± SD, *n* = 5. DGDG: digalactosyldiacylglycerol; MGDG: monogalactosyldiacylglycerol; LPA: lysophosphatidic acid; LPC: lysophosphatidylcholine; LPE: lysophosphatidylethanolamine; LPG: lysophosphatidylglycerol; MGMG: monogalactosylmonoacylglycerol; PA: phosphatidic acid; PC: phosphatidylcholine; PE: phosphatidylethanolamine; PG: phosphatidylglycerol; PI: phosphatidylinositol; PIP: phosphatidylinositol phosphate; PS: phosphatidylserine; CL: cardiolipin; SQDG: sulphoquinovosyldiacylglycerol

The levels of the total phospholipids, phosphatidic acid (PA), phosphatidylethanolamine (PE), phosphatidylserine (PS) and cardiolipin (CL) in *C. multipinnata* were significantly higher than those in *C. panzhihuaensis* under the control conditions (Figs. 3 and 5). Heat and species had significant interactive effects on the amounts of phosphatidylcholine (PC), PE and phosphatidylinositol phosphate (PIP) (Fig. 5). After heat stress, PC and PE increased significantly by 181.45% and 156.95% respectively in *C. panzhihuaensis* and PIP increased significantly by 725.94% in *C. multipinnata*. Compared to the corresponding control, phosphatidylinositol (PI) accumulated significantly in *C. multipinnata* and *C. panzhihuaensis,* by 89.11% and 52.59% respectively, after heat stress. Species had significant effects on PA, PI, PS, PIP, and CL. Although the amount of the total phospholipids did not differ significantly between the two species following heat stress (Fig. 3), the amounts of PA, PI, PIP, and CL were significantly greater in *C. multipinnata* than those in *C. panzhihuaensis* for the heat-stressed plants (Fig. 5). Among the main lipid species of the several lipid classes, there were significant interactive effects between species and heat stress on PA species including 35:4, 37:2, 37:4; PC species including 34:1, 34:2, 34:3, 36:1, 36:4, 36:5, 36:6 and 37:5; PE species including 34:3, 36:2, 36:3 and 36:4; PG species including 39:1 and 44:1; PI species including 34:1, 36:4, 36:6 and 50:2 (Additional file 3). The results indicated that the two species responded differently to heat stress in phospholipid composition.

Among the lipid classes of saccharolipids, only the level of digalactosyldiacylglycerol (DGDG) was significantly affected by heat and species (Fig. 5). For the control plants, the amount of DGDG in *C. multipinnata* was significantly higher than that in *C. panzhihuaensis*. Except that DGDG accumulated markedly in *C. panzhihuaensis*, none of the lipid classes of saccharolipids were affected in terms of amount by heat stress for both the species (Fig. 5). Moreover, none of the lipid classes of saccharolipids differed significantly in amount between *C. multipinnata* and *C. panzhihuaensis* following heat stress. The DGDG-MGDG ratio was not affected by species and heat stress and no significant interactive effects between the two factors were found (Additional file 4). Among the main lipid species of the several lipid classes, there were significant interactive effects between species and heat stress on MGMG 18:3; MGDG 36:6; DGDG 36:5 and 36:6; SQDG 40:8 (Additional file 5). Based on these results, heat stress had little effect on saccharolipid composition of *C. multipinnata* and *C. panzhihuaensis* and very few lipid classes and species of saccharolipid changed differently between the two species following heat stress.

The amount of lysophosphatidic acid (LPA) was only affected by species (Fig. 5). Compared to that of *C. panzhihuaensis*, the LPA level of *C. multipinnata* was significantly higher in both the control and heat-treated plants. The levels of lysophosphatidylcholine (LPC) and lysophosphatidylethanolamine (LPE) were significantly affected by heat and species, with significant interactive effects between the two factors. Compared to those of the control, the amounts of LPC and LPE increased by more than 7 and 11 times respectively in *C. multipinnata* and increased by more than 13 and 38 times respectively in *C. panzhihuaensis*. In comparison with those in *C. panzhihuaensis*, the amounts of both LPC and LPE were markedly lower in *C. multipinnata* following heat stress. The level of lysophosphatidylglycerol (LPG) was significantly affected by heat stress. After heat shock, LPG accumulated by more than 8 times in *C. multipinnata* and increased by more than 3 times in *C. panzhihuaensis*. The LPG level of *C. multipinnata* was significantly lower than that of *C. panzhihuaensis* in the control plants. However, LPG amount did not differ significantly between the two species in the heat-treated plants. Heat and species had significant interactive effects on LPC 16:0, 18:0, 18:1, 18:2 and 18:3; LPE 16:0, 18:0, 18:2 and 18:3 (Additional file 3). The results indicated that most of the lipid classes of lysophospholipids accumulated substantially in both the species after heat stress.

#### Effects of heat stress on the unsaturation level of glycerolipids

Heat had significant effects on the DBI of DAG, TAG, and the total neutral glycerolipids, which improved significantly following heat stress for both the species (Fig. 4). The DBI of TAG and the total neutral glycerolipids was significantly affected by species, being lower in *C. multipinnata*. Moreover, the unsaturation levels of DAG and TAG were significantly lower in *C. multipinnata* than those in *C. panzhihuaensis* after heat shock. The results showed that heat induced the increase of unsaturation level of DAG and TAG to a different extent in the two species.

**Fig. 6:**
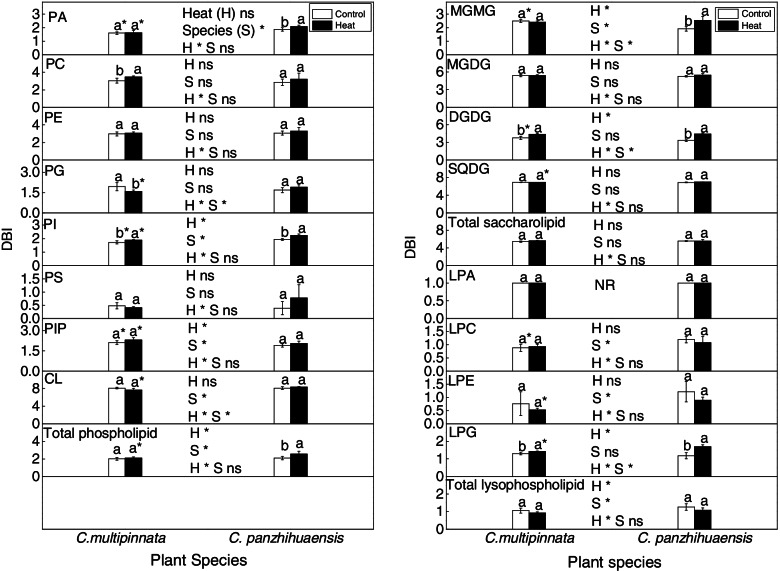
The DBI (double bond index) of each lipid class of phospholipids, saccharolipids and lysophospholipids following heat stress in *C. multipinnata* and *C. panzhihuaensis.* Two-way ANOVA analysis was performed in the general linear model. * indicates *P* ≤ 0.05 and ns indicates not significant. For the same species, different letters in the same column are significantly different between treatments according to independent samples T-test at *P* ≤ 0.05. For the same treatment, * is significantly different between species at *P* ≤ 0.05. Values shown are the mean ± SD, *n* = 5. DGDG: digalactosyldiacylglycerol; MGDG: monogalactosyldiacylglycerol; LPA: lysophosphatidic acid; LPC: lysophosphatidylcholine; LPE: lysophosphatidylethanolamine; LPG: lysophosphatidylglycerol; MGMG: monogalactosylmonoacylglycerol; PA: phosphatidic acid; PC: phosphatidylcholine; PE: phosphatidylethanolamine; PG: phosphatidylglycerol; PI: phosphatidylinositol; PIP: phosphatidylinositol phosphate; PS: phosphatidylserine; CL: cardiolipin; SQDG: sulphoquinovosyldiacylglycerol

The DBI of PI, PIP, and the total phospholipids was significantly affected by heat and species, with no interactive effects between the two factors (Fig. 6). Moreover, the DBI of both PA and CL was affected by species. The DBI of PC and PI increased significantly and that of phosphatidylglycerol (PG) decreased significantly after heat stress in *C. multipinnata*. However, the unsaturation level of its total phospholipids remained unchanged following heat stress. The DBI of PA, PI, and the total phospholipids increased markedly following heat stress in *C. panzhihuaensis*. In comparison with those in *C. panzhihuaensis*, the DBI of PA, PG, PI, CL, and the total phospholipids were significantly lower but that of PIP was significantly higher in *C. multipinnata* for the heat-stressed plants. Taken together, the two species were differentially affected by heat stress in the unsaturation level of membrane phospholipids.

The DBI of both monogalactosylmonoacylglycerol (MGMG) and DGDG was significantly affected by heat, with significant interactive effects between heat and species (Fig. 6). The DBI of MGMG and DGDG increased significantly in *C. panzhihuaensis* and that of DGDG also increased significantly in *C. multipinnata* after heat stress (Fig. 6). Heat and species had no effects on the DBI of monogalactosyldiacylglycerol (MGDG), sulphoquinovosyldiacylglycerol (SQDG), and the total saccharolipids, and no interactive effects between the two factors were found. However, the unsaturation level of SQDG was markedly lower in *C. multipinnata* than that in *C. panzhihuaensis* after heat shock. These results suggested that heat stress had little effect on the degree of unsaturation of the total membrane saccharolipids in the two species.

The DBI of LPC, LPE, and the total lysophospholipids differed significantly between the two species, being lower in *C. multipinnata* (Fig. 6). The DBI of LPG increased significantly after heat stress for both species, and that of other lipid classes of lysophospholipids and the total lysophospholipids remained unchanged (Fig. 6). The unsaturation levels of LPE and LPG were significantly lower in *C. multipinnata* than those in *C. panzhihuaensis* following heat stress. These results indicated that heat stress had little effect on the degree of unsaturation of the total lysophospholipids in the two species.

## Discussion

### The differential tolerance of *C. multipinnata* and *C. panzhihuaensis* to heat stress

The photosynthetic complexes in the thylakoid membranes are the key players in light reactions of photosynthesis but very sensitive to heat stress [46, 47]. The obvious decline of Fv/Fm (Table 1) shows that heat induced photoinhibition in both the species. Photoinhibition is associated with the photodamage or the enhanced thermal dissipation of PSII [48, 49]. As qN declined significantly in *C. multipinnata* and remained unchanged in *C. panzhihauensis* following heat shock and recovery, the induced photoinhibition was likely associated with the damage of PSII. This might be related to the accumulation of reactive oxygen species (ROS) which can block the repair of photodamaged PSII by suppressing the synthesis of proteins including D1 proteins [50]. The non-significant difference of Fv/Fm between the two species following heat stress indicates that the maximum photosynthetic potential of the two species was similar immediately after heat shock. However, according to Y(II), qP, and rETR, the photochemical reaction can still occur to some extent in *C. multipinnata,* but this process might be almost inhibited in *C. panzhihuaensis* under light conditions. This demonstrates that *C. multipinnata* was affected by heat shock to a lesser extent than *C. panzhihauensis*. qP reflects the openness of the reaction center of PSII [51].The values of Y(II), qP and rETR in *C. panzhihuaensis* decreased to near zero which indicated that the reaction center of PSII was closed after heat stress. As the chloroplast ultrastructure of *C. panzhihuaensis* were severely disordered, it might be due to the fact that the reaction center of PSII was inactivated or damaged and PSII repair process was inhibited [52]. In both the species, Y(II) and rETR were more sensitive to heat stress than Fv/Fm based on the degree of their decrease. The more sensitivity of Y(II) and rETR to stress than Fv/Fm was also found in some other studies [53, 54]. For example, Fv/Fm decreased slightly, but Y(II) decreased sharply by iron deficiency [54]. Y(NPQ) is photoprotective energy dissipation of PSII and Y(NO) is the fraction of energy that is passively dissipated which consists of qN due to photoinactivation of PSII and constitutive thermal dissipation [55, 56]. The significant decline of Y(NPQ) and the increase of Y(NO) demonstrate that plants of *C. panzhihuaensis* could not prevent photodamage through heat dissipation. Y(NO) also increased significantly following heat stress in *C. multipinnata*, which shows the decreased ability of the species to protect itself against damage by excess light energy. After 1 d of recovery, the significantly higher of Fv/Fm, Y(II), qP and rETR and significantly lower of Y(NO) in *C. multipinnata* confirmed that *C. multipinnata* was more tolerant to heat stress than *C. panzhihuaensis*.

Chloroplast as the site of photosynthesis is the main place of ROS production which is the most sensitive to abiotic stress [57, 58]. Therefore, the stability of chloroplast ultrastructure is closely associated with photosynthetic performance under adverse environmental conditions. High temperatures can damage the chloroplast and thylakoid membranes and disrupt the grana arrangement [13, 18, 59]. The chloroplast ultrastructures of either species were affected to different extents immediately following heat stress*.* This might be related to the cell position within the leaves and the developmental stage of chloroplasts. The more severely affected photosynthesis of *C. panzhihuaensis* by heat shock was partly due to the more damaged chloroplast ultrastructure of this species (Fig. 2).

Chlorophyll plays important roles in light capture, transfer and charge separation in photosynthesis [60]. High temperatures can lead to chlorophyll loss which is related to the heat sensitivity of plants [10, 61]. However, chlorophyll accumulated significantly in *C. panzhihuaensi*s after 1 d of recovery in comparison with the control (Fig. 1). Heat-induced accumulation of chlorophyll was also found in *Nouelia insignis* [62]. This phenomenon might be related to the decreased stability of chlorophyll-protein complexes which enables chlorophyll be more easily extracted [63]. Although the chlorophyll content did not decrease after 1 d of recovery, the leaf tip of some leaves of *C. multipinnata* turned yellow and most leaves of *C. panzhihuaensis* even bleached thereafter. This suggests that the adopted heat intensity was relatively severe to *C. multipinnata* and *C. panzhihuaensis* and the damaging effects of heat stress on these plants appeared progressively. The dynamic changes of leaf color in the two heat-stressed *Cycas* species suggest that the enzymes such as haem oxygenase which are involved in the pigment metabolism might be degraded or inactivated [64, 65].

The mean annual temperature in southwest China shows a marked rising trend [66]. The frequency and intensity of extreme heat events will be likely to increase in the habitats of the two species. The results of chlorophyll fluorescence and the chloroplast ultrastructures suggest that the increasing climate warming will be more detrimental to *C. panzhihuaensis* than *C. multipinnata.* Compared to *C. panzhihuaensis*, *C. multipinnata* distribute in the areas of lower latitudes and altitudes. The long-term adaptation to such habitats might confer higher thermotolerance to this species.

### The changes in neutral glycerolipids following heat stress

The lipid composition can affect plant stress tolerance through affecting membrane functioning and cellular processes [22, 67]. *C. multipinnata* and *C. panzhihuaensis* followed a similar change trend in terms of the amounts of most of the lipid categories after extreme heat stress (Fig. 3). This suggests that the two species have something in common in lipid adjustment to respond to heat stress.

As the main storage lipids, neutral glycerolipids accumulate in some plants under stress [68, 69]. Légeret et al. [69] have shown that the accumulated storage lipids under heat stress are converted from membrane lipids. Pick & Avidan [70] have demonstrated that TAG is produced from starch and polar lipids in the green alga *Dunaliella tertiolecta* under nitrogen deprivation. As no other lipids are degraded in the two species and there exists cross-talk between metabolisms of carbohydrates and lipids in the regulation of energy homeostasis [71], heat might have triggered the increased carbon flow to fatty acid synthesis pathways in the two species. The accumulated neutral glycerolipids in *C. multipinnata* and *C. panzhihuaensis* following heat shock (Fig. 4) may act as a source of energy until the photosynthetic apparatus is repaired. The role of neutral glycerolipids is not limited to their storage function. It has been found that DAG plays crucial roles in signal transduction and TAG is conducive to stabilizing membranes, protecting cells against photodamage and consuming excessive photoassimilates [68, 72]. However, DAG is an amphiphilic, non-bilayer-forming lipid and incorporation of DAG in membranes increases the tendency of membranes to form H_II_-hexagonal phases and change the membrane properties [73]. Therefore, the conversion of DAG to TAG can increase the membrane stability and augment plant thermotolerance [74]. The marked decline of DAG/TAG and the significantly lower ratio in *C. multipinnata* than that in *C. panzhihuaensis* after heat stress (Fig. 4) would be more beneficial to the maintenance of membrane stability for the species [72, 74].

Although TAG generally accumulates in response to stress conditions, the unsaturation level of the accumulated TAG varies with plant species and stress type [69, 75]. It has been shown that heat initiated the accumulation of polyunsaturated TAG in wheat (*Triticum aestivum* L.) and unicellular green algae (*Chlamydomonas reinhardtii*) [24, 69]. However, viral infection and nitrogen starvation mainly induce the production of more saturated TAG in some algae [75, 76]*.* It has been suggested that the newly accumulated polyunsaturated TAG under heat stress was formed via direct conversion of MGDG [69]. Therefore, TAG is involved in the heat acclimation by sequestering the polyunsaturated fatty acid from the membrane lipids [24]. As the amount and unsaturation level of each class of saccharolipids remained unchanged or increased (Fig. 6), the increased unsaturated storage lipids (Fig. 4) are not likely converted from saccharolipids. The mechanisms of increased unsaturation level of neutral glycerolipids in both *C. multipinnata* and *C. panzhihuaensis* following heat stress need to be further verified.

### The composition of membrane glycerolipids following heat stress

Membrane properties and the functioning of membrane proteins are regulated by lipid composition [67, 77]. However, phospholipids and saccharolipids tend to be degraded under environmental stress [23, 78, 79]. For *C. multipinnata* and *C. panzhihuaensis*, each lipid class of phospholipids and saccharolipids remained unchanged or increased in amount immediately following heat stress. This suggests that they may have positively coped with the extreme high temperature through stabilizing the membrane systems. Phospholipids were more responsive to heat stress than saccharolipids in terms of amounts in both species, particularly in *C. panzhihuaensis* (Fig. 3). This demonstrates that heat stress has imposed differential effects on plastidic and extraplastidic membranes. Some other studies have also suggested that the two types of membranes differentially respond to environmental stress [23, 80]. This phenomenon might be related to the differential position, composition, and structure between the two types of membranes.

The ratio of PC to PE affects the membrane stability [22]. The proportional increase of PC and PE under stress in *C. panzhihuaensis* (Fig. 5) might be beneficial to maintain the membrane stability or repair the damaged membrane. PI and PIP are the components of the PI signal system, which play key roles in the perception and transduction of environmental stimuli [81]. The significant increase of PI and PIP in *C. multipinnata* and the increase of PI in *C. panzhihuaensis* (Fig. 5) implies that the PI signal pathway might participate in the response and adaptation of the two species to heat stress. PA is an intermediate in glycerolipid metabolism and an important signaling molecule [82]. CL plays an important role in a variety of mitochondrial events [83, 84]. Whether the significantly higher amounts of PA, PI, PIP, and CL under heat stress are related to the greater thermotolerance of *C. multipinnata* needs to be verified. The membrane fluidity is crucial to ensure the proper cellular processes associated with membranes [22]. The significant increase of unsaturation level in PC and PI of *C. multipinnata* and in PA, PI, and total phospholipids of *C. panzhihuaensis* (Fig. 6) might induce changes in the physical and chemical properties of extraplastidic membranes, which can adversely affect the cellular processes. However, the significantly lower unsaturation level of PA, PI, CL, and the total phospholipids in *C. multipinnata* than that in *C. panzhihuaensis* could make the former possess more stable membranes and higher heat tolerance.

Thylakoids are the most abundant membranes of leaf tissues in which light harvesting and photosynthetic energy conversion take place [85]. Therefore, adjustment in the composition and the resultant changes of fluidity of thylakoid membranes could affect the adaptability of plants to heat stress. It is generally recognized that plants decrease the lipid unsaturation level to maintain a stable membrane under heat stress [24, 69]. The increase of DGDG amount following heat stress (Fi. 5) might be a defense strategy of *C. panzhihuaensis* to alleviate the heat damage to photosynthetic membranes. However, the significant increase of the unsaturation level of MGMG and DGDG in *C. panzhihuaensis* and the increase of the unsaturation level of DGDG in *C. multipinnata* (Fig. 6) might have induced changes in the properties of plastidic membranes, which led to the decrease of the photosynthetic activity. Besides the several lipid classes mentioned above, PG and SQDG are also important constituents of chloroplast membranes [86, 87]. The unsaturation level of PG decreased markedly under heat stress in *C. multipinnata* (Fig. 6), which could be conducive to maintaining the membrane integrity and functions. The significantly lower unsaturation degrees of SQDG and PG in *C. multipinnata* than those in *C. panzhihuaensis* might contribute to the higher stability of chloroplast membranes and higher photosynthetic activity of the species under heat stress.

Lysophospholipids are implicated in the maintenance of the membrane skeleton and cell signaling associated with growth, development, and stress-related response [20]. However, the substantial accumulation of lysophospholipids in both the species (Fig. 5) might result in membrane fusion and cell death [88, 89]. Compared to *C. multipinnata*, the greater percentage of increase and the higher levels of lysophospholipids after heat stress might be related to the weaker thermotolerance of *C. panzhihuaensis*. Besides, the difference between the two species in the unsaturation level of LPE and LPG (Fig. 6) might also contribute to their differential response to heat shock.

### The changes in sterol lipids and wax esters following heat stress

Sterol lipids are structural components of cell membranes which can sustain and reinforce the domain structure by regulating acyl chain ordering [90]. Meanwhile, they play critical roles in fundamental metabolic and developmental processes [91]. Therefore, sterol lipids are expected to be involved in plant response to stresses. High temperatures tend to increase membrane fluidity. The significant accumulation of sterol lipids under heat stress in both the species (Fig. 3) was conducive to the maintenance of membrane fluidity and integrity. This might be a common defense strategy to cope with extreme high temperature for *C. multipinnata* and *C. panzhihuaensis*. Surface protection through deposition of cuticular wax is also a crucial function of lipids to resist stresses [92, 93]. Studies have shown that wax amount and composition will change to improve plant adaptation to changing environments [94]. Wax esters generally represent a small part of wax components on the plant surfaces [95]. It has been shown that wax esters increase during water deficiency, which contributes to the drought tolerance of *Arabidopsis* [95]. The substantial deposition of wax esters in both the species under heat stress (Fig. 3) might be beneficial for improving heat tolerance by reducing water loss. Meanwhile, the much more drastic increase and greater deposition of wax esters might enable *C. multipinnata* to be more tolerant to heat stress than *C. panzhihuaensis*. The findings suggest that wax esters might be potential molecular markers for heat tolerance, which however, needs to be confirmed by further studies.

## Conclusions

The results of chlorophyll fluorescence parameters and chloroplast ultrastructure indicate that *C. multipinnata* is more tolerant to extreme high temperature than *C. panzhihuaensis*. *C. multipinnata* and *C. panzhihuaensis* followed a similar change trend in the amounts of most lipid categories after heat stress. The amounts of all the lipid categories except lysophospholipids and fatty acyls (wax esters) did not differ significantly between the two species following heat shock. The much more drastic increase of lysophospholipids and wax esters in *C. panzhihuaensis* and *C. multipinnata* respectively might contribute to the differentiation of heat tolerance of the two species. Meanwhile, the significantly lower unsaturation level of SQDG, PA, PG, PI, CI, LPE, and LPG in *C. multipinnata* might make the species more tolerant to heat. The degree of heat effects are affected by many factors such as the heat intensity, the speed and duration of the temperature increase, and other environmental conditions [9, 96]. Based on our observations, the damaging symptoms of heat stress appeared progressively. Therefore, further studies will be conducted to explore the responses of *Cycas* species to different types of heat stress and their post-heat recovery performance. Moreover, the molecular mechanisms of the differential thermotolerance of photosynthesis between the two species should be explored. Heat tolerance of *C. panzhihuaensis* might be improved by modulating photosynthesis through genetic engineering [97]*.* Our study and observations show that *C. panzhihuaensis* is more tolerant to freezing temperatures than several other *Cycas* species [98]. Therefore, introduction of this species in areas of higher latitudes and altitudes might be also an effective way to cope with the increasing temperature.

## Methods

### Plant growth and treatments

Seeds of *C. panzhihuaensis* were collected in Panzhihua. This was approved by the Administration Bureau of Panzhihua Cycas National Nature Reserve, Sichuan province. Seeds of *C. multipinnata* were collected from horticultural sources in Gejiu, Yunnan province. Seed collection was permitted by private land owners. The seeds of *C. multipinnata* and *C. panzhihuaensis* were sown on a moist sand bed in Southwest Forestry University. After emergence, the seedlings were transplanted in individual plastic pots (20 cm in diameter and 14 cm in height) with a mixture of sand, humus, and laterite soil (1:1:1) and cultivated in a culture room where the temperature, humidity and light intensity can not be automatically controlled. The seedlings were watered about every 3–5 days according to conditions, and each pot received 2 g compound fertilizer (N-P_2_O_5_-K_2_O: 18–10-12) every 3 months. The germinated five-year-old seedlings of *C. multipinnata* and *C. panzhihuaensis* were grown in a greenhouse in Southwest Forestry University and used to conduct the experiments. The seedlings of the two species were identified by Shuangzhi Li, a taxonomist expert at Southwest Forestry University. Voucher specimens of *C. panzhihuaensis* and *C. multipinnata* have been deposited in the herbarium of Southwest Forestry University with an accession number No. ZYL-001 and No. ZYL-005, respectively.

**Fig. 7:**
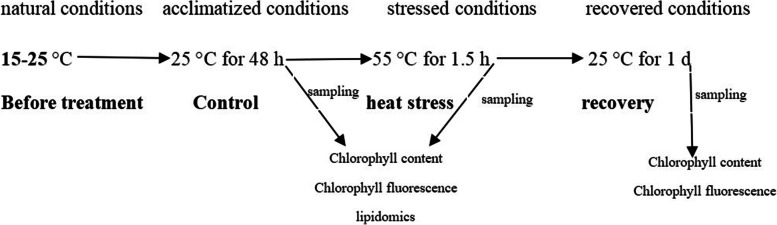
The outline of heat stress experiment. Temperatures were shown for each of the four phases. Sampling represents sampling time points for specific measurements at the ends of phases

The temperature of the culture room was about 15–25 °C and the daytime maximum photosynthetic photon flux density was approximately 600 μmol m^−2^ s^−1^ during the period we conducted the experiment. The temperature during the experiment was shown in Fig. 7. Plants of both the species were acclimatized for 48 h at 25 °C (suitable temperature for their growth) in a growth chamber with a light intensity of 600 μmol m^−2^ s^−1^ and a 12-h photoperiod. These plants were used as the control. Based on our previous study, the photosynthetic activity of *C. multipinnata* was not affected by heat shock at 25–45 °C for 2 h but significantly impacted by heat shock at 55 °C for 2 h [99]. Considering the possible lower thermotolerance of *C. panzhihuaensis* in comparison with *C. multipinnata*, seedlings of the two species were heat shocked at 55 °C for 1.5 h. After heat shock, one part of plants were used to measure physiological and biochemical characteristics, and the other part of plants were recovered for 1 d at the acclimatized conditions for measurement of chlorophyll content and chlorophyll fluorescence. Immediately following treatments, leaves of some plants were sampled, frozen in liquid nitrogen, and then stored at -80 °C for further measurement of chlorophyll content and lipid compositions.

### Measurements of chlorophyll fluorescence

Chlorophyll fluorescence was measured on attached leaves with a chlorophyll fluorometer (PAM-2500, Walz, Germany) following 30 min of dark adaptation. The saturating light pulse was set at about 8000 µmol m^–2^ s^–1^ for 0.8 s. The actinic light was set at 617 µmol m^–2^ s^–1^ and the plants were adapted to such actinic light for more than 5 min. The Foʹ measuring mode was activated for measurement of Foʹ. This means that a 5 s interval follows each saturation pulse during which the actinic light is switched off and far-red illumination is turned on. Maximum quantum yield of PSII (Fv/Fm), actual photochemical quantum production of PSII (Y(II)), photochemical quenching coefficient (qP), non-photochemical quenching coefficient (qN), quantum yield of non-regulated (Y(NO)) and regulated (Y(NPQ)) non-photochemical energy dissipation, and relative electron transport rate (rETR) were measured.

### Measurement of the chloroplast ultrastructure

The ultrastructure of the leaf cells was measured according to the methods described by Liu et al. [100], with minor modification. Leaf samples were cut into small pieces (about 1 cm × 1 cm) and fixed in 2.5% glutaraldehyde solution at 4 °C. The samples were washed five times with 0.1 mol L^−1^ phosphate buffer and fixed overnight in 1% osmic acid at 4 °C. The materials were dehydrated using a series of acetone solutions (50%, 70% and 90%, v/v), for 15 min at each concentration, and then dehydrated with absolute acetone three times, for 15–20 min each time. The materials were then infiltrated with acetone and resin (EPon812) at proportions of 2:1 (v/v) for 0.5 h at room temperature and at proportions of 1:2 (v/v) for 1.5 h at 37 °C, followed by infiltration with 100% resin for 3 h at 37 °C. After separate polymerization at 37, 45, and 60 °C for 24 h in turn, thin sections were created using an ultramicrotome (Reicher-Jung ULTRACUT, Austria) and the sections were double-stained with uranyl acetate–lead citrate. We examined the samples using a model JEM1200 transmission electron microscope (JEOL, Tokyo, Japan).

### Chlorophyll content determination

Foliar chlorophyll was extracted with 95% ethanol and absorbance was measured using a spectrophotometer (Shimadzu uv-2450) at 665 nm and 649 nm to determine the concentration of chlorophyll *a* and chlorophyll *b*. The content of chlorophyll (a + b) was calculated according to Wang [101].

### Lipidome analysis

The process of lipid extraction and mass spectrometry-based lipid detection was described in detail by Zhu et al. [102]. In brief, the frozen sample was ground to powder in liquid nitrogen and then homogenized with the solution including 200 µL distilled water, 240 µL pre-cooling methanol, and 800 µL methyl tert-butyl ether. The total lipid extract was dried under a gentle stream of nitrogen. The sample was dissolved in 100 µL isopropanol and analyzed with an ultra-high-performance liquid chromatography (UHPLC) Nexera LC-30A C18 column (100 mm × 2.1 mm, 1.7 µm) at 45 °C. Full-scan spectra were collected in mass-to-charge ratio (*m*/*z*) ranges of 200–1800 and 250–1800 for positive and negative ion modes, respectively. The mass-to-charge ratio of lipid molecules to lipid fragments was collected by the following method: after each full scan, 10 fragment patterns (MS2 scan, HCD) were collected. Lipid identification (secondary identification), peak extraction, peak alignment, and quantification were assessed with LipidSearch software version 4.1 (ThermoFisher Scientific Inc, Waltham, MA, USA).

### Calculation of lipid double bond index (DBI)

The DBI was calculated according to the method described by Zheng et al. [103]. DBI = (∑[*N* × mol % lipid])/100, where *N* is the number of double bonds in each lipid molecule.

### Statistical analysis

Five replicates for chlorophyll fluorescence parameters, chlorophyll content and lipidomics were arranged for each treatment. Measurement of chlorophyll fluorescence was performed on one leaf of each plant for each replicate, and sample collection for analysis of chlorophyll content and lipidomics was performed from three plants for each replicate. The data from discordant samples were removed based on the Q-test [90]. All data were analyzed by two-way analysis of variance (ANOVA) using the general linear model (GLM) in SPSS 15.0 software (IBM Corp., Armonk, NY). Comparisons between treatments within one species and between the two species within one treatment were evaluated by the independent sample T-test or one-way ANOVA (*P* ≤ 0.05) depending on the number of factor levels. The results are presented as the mean ± SD.

## Supplementary Information

**Additional file 1.** The leaf morphological characteristics of *Cycas multipinnata* and *C. panzhihuaensis* subjected to control conditions and 7 d of recovery from heat stress.

**Additional file 2.** All the identified lipid species in leaves of *Cycas multipinnata* and *C. panzhihuaensis.*

**Additional file 3.** Changes in lipid molecular species of glycerophospholipids and lysophospholipids in *Cycas multipinnata* and *C. panzhihuaensis *subjected to heat stress.

**Additional file 4.** The digalactosyldiacylglycerol (DGDG)-monogalactosyldiacylglycerol (MGDG) ratio of *Cycas multipinnata* and *C. panzhihuaensis *subjected to heat stress*.*

**Additional file 5.** Changes in lipid molecular species of saccharolipids in *Cycas multipinnata* and *C. panzhihuaensis *subjected to heat stress.

## Data Availability

The datasets used and/or analyzed during the current study are available from the corresponding author on reasonable request.

## Abbreviations

ACL: Acyl chain length
CL: Cardiolipin
DAG: Diacylglycerol
DBI: Double bond index
DGDG: Digalactosyldiacylglycerol
Fv/Fm: The maximum quantum yield of photosystem II (PSII)
MGDG: Monogalactosyldiacylglycerol
MGMG: Monogalactosylmonoacylglycerol
NPQ: Non-photochemical quenching coefficient
PA: Phosphatidic acid
PC: Phosphatidylcholine
PE: Phosphatidylethanolamine
PG: Phosphatidylglycerol
PI: Phosphatidylinositol
PIP: Phosphatidylinositol
PS: Phosphatidylserine
qP: Photochemical quenching coefficient
rETR: Relative electron transport rate
SQDG: Sulphoquinovosyldiacylglycerol
TAG: Triacylglycerol
Y(II): Effective quantum yield of PS II
Y(NO): Non-regulated non-photochemical energy loss in PS II
Y(NPQ): Regulated non-photochemical energy loss in PS II
