# Supplementary material for: Effects of heat shock on photosynthesis-related characteristics and lipid profile of Cycas multipinnata and C. panzhihuaensis
Source: BMC Plant Biol. 2022 Sep 15;22:442. doi: 10.1186/s12870-022-03825-0 (PMC9476270; doi:10.1186/s12870-022-03825-0)
Supplement: Supplementary file 1 — Additional file 1. The leaf morphological characteristics of Cycas multipinnata and C. panzhihuaensis subjected to control conditions and 7 d of recovery from heat stress. [file 12870_2022_3825_MOESM1_ESM.docx]

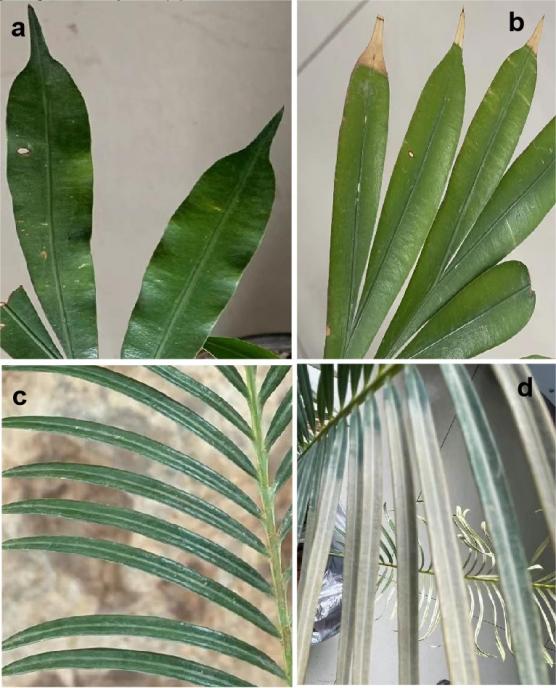


**Additional file 1.** The leaf morphological characteristics of *Cycas multipinnata* and *C. panzhihuaensis* subjected to control conditions and 7 d of recovery from heat stress. a and b show the leaf morphological characteristics of the control and recovered plants in *C. multipinnata*, respectively; c and d show the leaf morphological characteristics of the control and recovered plants in *C. panzhihuaensis*, respectively.
