# Supplementary material for: Effects of heat shock on photosynthesis-related characteristics and lipid profile of Cycas multipinnata and C. panzhihuaensis
Source: BMC Plant Biol. 2022 Sep 15;22:442. doi: 10.1186/s12870-022-03825-0 (PMC9476270; doi:10.1186/s12870-022-03825-0)
Supplement: Supplementary file 2 — Additional file 2. All the identified lipid species in leaves of Cycas multipinnata and C. panzhihuaensis. [file 12870_2022_3825_MOESM2_ESM.docx]

**Additional file 2.** All the identified lipid species in leaves of *Cycas multipinnata* and *C. panzhihuaensis.*

| **Lipid class** | **Lipid class (abbreviation)** | **Lipid species** | **Calmz** | **Formula** | **RT-(min)** |
| --- | --- | --- | --- | --- | --- |
| Ceramides | Cer | Cer(d34:3)+H | 534.4880705 | C34 H64 O3 N1 | 10.20004177 |
|  | Cer | Cer(d38:2+O)+H | 608.5612355 | C38 H74 O4 N1 | 12.25605406 |
|  | Cer | Cer(d40:1+hO+O)+H | 654.6031005 | C40 H80 O5 N1 | 12.90959066 |
|  | Cer | Cer(d42:1+hO)+H | 666.6394855 | C42 H84 O4 N1 | 14.4346708 |
|  | Cer | Cer(d41:1+hO+O)+H | 668.6187505 | C41 H82 O5 N1 | 13.43977169 |
|  | Cer | Cer(d42:0+pO)+H | 668.6551355 | C42 H86 O4 N1 | 15.01407694 |
|  | Cer | Cer(d43:1+O)+H | 680.6551355 | C43 H86 O4 N1 | 15.02774313 |
|  | Cer | Cer(d42:1+hO+O)+H | 682.6344005 | C42 H84 O5 N1 | 13.9795455 |
|  | Cer | Cer(d44:1+hO)+H | 694.6707855 | C44 H88 O4 N1 | 15.62917523 |
|  | Cer | Cer(d47:7+hO)+H | 724.6238355 | C47 H82 O4 N1 | 15.61620852 |
|  | Cer | Cer(d34:2+O)+HCOO | 596.4895625 | C35 H66 O6 N1 | 10.17958877 |
|  | Cer | Cer(d36:2+hO)+HCOO | 624.5208625 | C37 H70 O6 N1 | 11.25804749 |
|  | Cer | Cer(d39:0+pO+O)+HCOO | 686.5940275 | C40 H80 O7 N1 | 12.89533318 |
|  | Cer | Cer(d43:1+hO)+HCOO | 724.6460625 | C44 H86 O6 N1 | 15.02307295 |
|  | Cer | Cer(d40:1+hO)+HCOO | 682.5991125 | C41 H80 O6 N1 | 13.3045796 |
|  | Cer | Cer(d42:2+O)+HCOO | 708.6147625 | C43 H82 O6 N1 | 14.37948932 |
|  | Cer | Cer(d44:2+O)+HCOO | 736.6460625 | C45 H86 O6 N1 | 15.57096931 |
|  | Cer | Cer(d19:0)+H | 330.3002705 | C19 H40 O3 N1 | 1.92107882 |
|  | Cer | Cer(d25:0)+H | 414.3941705 | C25 H52 O3 N1 | 5.379612587 |
|  | Cer | Cer(d33:0)+H | 526.5193705 | C33 H68 O3 N1 | 10.09467004 |
|  | Cer | Cer(d33:0)+H | 526.5193705 | C33 H68 O3 N1 | 11.68820621 |
|  | Cer | Cer(d34:3)+H | 534.4880705 | C34 H64 O3 N1 | 9.416842362 |
|  | Cer | Cer(d34:2+O)+H | 552.4986355 | C34 H66 O4 N1 | 9.415593588 |
|  | Cer | Cer(d35:0)+H | 554.5506705 | C35 H72 O3 N1 | 12.93558853 |
|  | Cer | Cer(d35:0)+H | 554.5506705 | C35 H72 O3 N1 | 11.38173097 |
|  | Cer | Cer(d36:3)+H | 562.5193705 | C36 H68 O3 N1 | 10.55355357 |
|  | Cer | Cer(d34:1+2O)+H | 570.5092005 | C34 H68 O5 N1 | 7.756 |
|  | Cer | Cer(d36:2+O)+H | 580.5299355 | C36 H70 O4 N1 | 10.55188321 |
|  | Cer | Cer(d38:3)+H | 590.5506705 | C38 H72 O3 N1 | 11.54174984 |
|  | Cer | Cer(d38:2+O)+H | 608.5612355 | C38 H74 O4 N1 | 11.53718827 |
|  | Cer | Cer(d40:3)+H | 618.5819705 | C40 H76 O3 N1 | 12.49086306 |
|  | Cer | Cer(d40:0)+H | 624.6289205 | C40 H82 O3 N1 | 14.47842208 |
|  | Cer | Cer(d40:0+pO)+H | 640.6238355 | C40 H82 O4 N1 | 13.86537669 |
|  | Cer | Cer(d42:3)+H | 646.6132705 | C42 H80 O3 N1 | 13.50396888 |
|  | Cer | Cer(d41:0+pO)+H | 654.6394855 | C41 H84 O4 N1 | 14.41547919 |
|  | Cer | Cer(d40:0+pO+O)+H | 656.6187505 | C40 H82 O5 N1 | 13.43607701 |
|  | Cer | Cer(d42:2+O)+H | 664.6238355 | C42 H82 O4 N1 | 13.50458078 |
|  | Cer | Cer(d41:0+pO+O)+H | 670.6344005 | C41 H84 O5 N1 | 13.97422711 |
|  | Cer | Cer(d43:0+pO)+H | 682.6707855 | C43 H88 O4 N1 | 15.61103096 |
|  | Cer | Cer(d42:0+pO+O)+H | 684.6500505 | C42 H86 O5 N1 | 14.55089486 |
|  | Cer | Cer(d43:1+hO+O)+H | 696.6500505 | C43 H86 O5 N1 | 14.55811322 |
|  | Cer | Cer(d44:0+pO)+H | 696.6864355 | C44 H90 O4 N1 | 16.22909959 |
|  | Cer | Cer(d46:5)+H | 698.6445705 | C46 H84 O3 N1 | 18.702 |
|  | Cer | Cer(d43:0+pO+O)+H | 698.6657005 | C43 H88 O5 N1 | 15.13479154 |
|  | Cer | Cer(d47:7)+H | 708.6289205 | C47 H82 O3 N1 | 16.80653198 |
|  | Cer | Cer(d44:1+hO+O)+H | 710.6657005 | C44 H88 O5 N1 | 15.14225433 |
|  | Cer | Cer(d45:0+pO)+H | 710.7020855 | C45 H92 O4 N1 | 16.86839312 |
|  | Cer | Cer(d44:0+pO+O)+H | 712.6813505 | C44 H90 O5 N1 | 15.73999027 |
|  | Cer | Cer(d45:0+pO+O)+H | 726.6970005 | C45 H92 O5 N1 | 16.35812538 |
|  | Cer | Cer(d49:6)+H | 738.6758705 | C49 H88 O3 N1 | 18.16671997 |
|  | Cer | Cer(d49:6)+H | 738.6758705 | C49 H88 O3 N1 | 16.49990967 |
|  | Cer | Cer(d39:0+pO)+HCOO | 670.5991125 | C40 H80 O6 N1 | 13.28718633 |
|  | Cer | Cer(d42:0)+HCOO | 696.6511475 | C43 H86 O5 N1 | 16.141 |
|  | Cer | Cer(d40:1+pO+O)+HCOO | 698.5940275 | C41 H80 O7 N1 | 12.90288026 |
|  | Cer | Cer(d45:1+hO)+HCOO | 752.6773625 | C46 H90 O6 N1 | 16.24910108 |
|  | Cer | Cer(d46:0+pO)+HCOO | 768.7086625 | C47 H94 O6 N1 | 17.50585835 |
|  | Cer | Cer(d46:1+hO+O)+HCOO | 782.6879275 | C47 H92 O7 N1 | 16.3644568 |
|  | Cer | Cer(d25:0)+H | 414.3941705 | C25 H52 O3 N1 | 3.778266178 |
|  | Cer | Cer(d38:0+pO)+H | 612.5925355 | C38 H78 O4 N1 | 12.75853353 |
|  | Cer | Cer(d41:1+hO)+H | 652.6238355 | C41 H82 O4 N1 | 13.88114958 |
|  | Cer | Cer(d42:1+hO+O)+H | 682.6344005 | C42 H84 O5 N1 | 13.13794793 |
|  | Cer | Cer(d44:1+hO+O)+H | 710.6657005 | C44 H88 O5 N1 | 14.20146143 |
|  | Cer | Cer(d47:5+pO)+H | 728.6551355 | C47 H86 O4 N1 | 13.059 |
|  | Cer | Cer(d46:1+hO+O)+H | 738.6970005 | C46 H92 O5 N1 | 15.29664228 |
|  | Cer | Cer(d46:1+hO)+HCOO | 766.6930125 | C47 H92 O6 N1 | 16.89042315 |
| Simple Glc series | CerG1 | CerG1(d34:3+pO)+H | 712.5358105 | C40 H74 O9 N1 | 8.476966765 |
|  | CerG1 | CerG1(d34:2+O)+H | 714.5514605 | C40 H76 O9 N1 | 9.414419375 |
|  | CerG1 | CerG1(d36:3)+H | 724.5721955 | C42 H78 O8 N1 | 10.55159498 |
|  | CerG1 | CerG1(d36:2+O)+H | 742.5827605 | C42 H80 O9 N1 | 10.55190392 |
|  | CerG1 | CerG1(d38:3)+H | 752.6034955 | C44 H82 O8 N1 | 11.54523658 |
|  | CerG1 | CerG1(d37:2+O)+H | 756.5984105 | C43 H82 O9 N1 | 11.06365155 |
|  | CerG1 | CerG1(d38:2+O)+H | 770.6140605 | C44 H84 O9 N1 | 11.54024044 |
|  | CerG1 | CerG1(d40:3)+H | 780.6347955 | C46 H86 O8 N1 | 12.49502262 |
|  | CerG1 | CerG1(d41:3)+H | 794.6504455 | C47 H88 O8 N1 | 12.9891137 |
|  | CerG1 | CerG1(d40:2+O)+H | 798.6453605 | C46 H88 O9 N1 | 12.47655597 |
|  | CerG1 | CerG1(d42:3)+H | 808.6660955 | C48 H90 O8 N1 | 13.5035587 |
|  | CerG1 | CerG1(d41:2+O)+H | 812.6610105 | C47 H90 O9 N1 | 12.98939831 |
|  | CerG1 | CerG1(d43:3)+H | 822.6817455 | C49 H92 O8 N1 | 14.05134554 |
|  | CerG1 | CerG1(d42:2+O)+H | 826.6766605 | C48 H92 O9 N1 | 13.50470986 |
|  | CerG1 | CerG1(d44:3)+H | 836.6973955 | C50 H94 O8 N1 | 14.59713022 |
|  | CerG1 | CerG1(d43:2+O)+H | 840.6923105 | C49 H94 O9 N1 | 14.04997199 |
|  | CerG1 | CerG1(d43:1+hO)+H | 842.7079605 | C49 H96 O9 N1 | 13.91521589 |
|  | CerG1 | CerG1(d46:2+O)+H | 882.7392605 | C52 H100 O9 N1 | 15.77112978 |
|  | CerG1 | CerG1(d34:3)+H | 696.5408955 | C40 H74 O8 N1 | 9.413573638 |
|  | CerG1 | CerG1(d39:2+O)+H | 784.6297105 | C45 H86 O9 N1 | 12.02528098 |
|  | CerG1 | CerG1(d40:1+hO+O)+H | 816.6559255 | C46 H90 O10 N1 | 12.14774739 |
|  | CerG1 | CerG1(d41:1+hO+O)+H | 830.6715755 | C47 H92 O10 N1 | 12.64193866 |
|  | CerG1 | CerG1(d42:1+hO+O)+H | 844.6872255 | C48 H94 O10 N1 | 13.14049639 |
|  | CerG1 | CerG1(d45:3)+H | 850.7130455 | C51 H96 O8 N1 | 15.14716102 |
|  | CerG1 | CerG1(d44:2+O)+H | 854.7079605 | C50 H96 O9 N1 | 14.59858525 |
|  | CerG1 | CerG1(d46:1+hO+O)+H | 900.7498255 | C52 H102 O10 N1 | 15.32029503 |
| Sphingosine | So | So(d18:1)+H | 300.2897055 | C18 H38 O2 N1 | 3.661645015 |
|  | So | So(d17:1)+H | 286.2740555 | C17 H36 O2 N1 | 2.061258598 |
|  | So | So(d17:1)+H | 286.2740555 | C17 H36 O2 N1 | 2.457961585 |
|  | So | So(d17:0+pO)+H | 304.2846205 | C17 H38 O3 N1 | 1.876286553 |
|  | So | So(d18:0+pO)+H | 318.3002705 | C18 H40 O3 N1 | 2.3716643 |
| Sphingomyelin | SM | SM(d22:1+hO)+H | 551.3819675 | C27 H56 O7 N2 P1 | 2.824007807 |
| Phosphatidic acid | PA | PA(32:3)-H | 641.4187815 | C35 H62 O8 N0 P1 | 6.929 |
|  | PA | PA(33:1)-H | 659.4657315 | C36 H68 O8 N0 P1 | 10.47924683 |
|  | PA | PA(33:0)-H | 661.4813815 | C36 H70 O8 N0 P1 | 10.8607364 |
|  | PA | PA(34:3)-H | 669.4500815 | C37 H66 O8 N0 P1 | 12.12154034 |
|  | PA | PA(34:2)-H | 671.4657315 | C37 H68 O8 N0 P1 | 12.06899141 |
|  | PA | PA(34:1)-H | 673.4813815 | C37 H70 O8 N0 P1 | 12.10547792 |
|  | PA | PA(34:1)-H | 673.4813815 | C37 H70 O8 N0 P1 | 11.67609831 |
|  | PA | PA(35:4)-H | 681.4500815 | C38 H66 O8 N0 P1 | 8.98784704 |
|  | PA | PA(35:3)-H | 683.4657315 | C38 H68 O8 N0 P1 | 9.441103413 |
|  | PA | PA(35:3)-H | 683.4657315 | C38 H68 O8 N0 P1 | 9.741458 |
|  | PA | PA(35:2)-H | 685.4813815 | C38 H70 O8 N0 P1 | 10.15738304 |
|  | PA | PA(35:1)-H | 687.4970315 | C38 H72 O8 N0 P1 | 10.911884 |
|  | PA | PA(35:1)-H | 687.4970315 | C38 H72 O8 N0 P1 | 11.4017947 |
|  | PA | PA(35:0)-H | 689.5126815 | C38 H74 O8 N0 P1 | 11.78392276 |
|  | PA | PA(36:3)-H | 697.4813815 | C39 H70 O8 N0 P1 | 11.31608681 |
|  | PA | PA(36:3)-H | 697.4813815 | C39 H70 O8 N0 P1 | 11.01282659 |
|  | PA | PA(36:2)-H | 699.4970315 | C39 H72 O8 N0 P1 | 11.67027169 |
|  | PA | PA(36:1)-H | 701.5126815 | C39 H74 O8 N0 P1 | 11.34264397 |
|  | PA | PA(37:4)-H | 709.4813815 | C40 H70 O8 N0 P1 | 9.502381982 |
|  | PA | PA(37:2)-H | 713.5126815 | C40 H74 O8 N0 P1 | 11.03218221 |
|  | PA | PA(37:1)-H | 715.5283315 | C40 H76 O8 N0 P1 | 11.81983492 |
|  | PA | PA(44:2)-H | 811.6222315 | C47 H88 O8 N0 P1 | 12.60244061 |
|  | PA | PA(32:0)-H | 647.4657315 | C35 H68 O8 N0 P1 | 11.78680937 |
|  | PA | PA(36:5)-H | 693.4500815 | C39 H66 O8 N0 P1 | 9.446401353 |
|  | PA | PA(43:2)-H | 797.6065815 | C46 H86 O8 N0 P1 | 10.62901449 |
|  | PA | PA(44:3)-H | 809.6065815 | C47 H86 O8 N0 P1 | 15.67176737 |
| Phosphatidylcholine | PC | PC(32:1)+H | 732.5537835 | C40 H79 O8 N1 P1 | 11.12 |
|  | PC | PC(34:3)+H | 756.5537835 | C42 H79 O8 N1 P1 | 9.644959277 |
|  | PC | PC(34:2)+H | 758.5694335 | C42 H81 O8 N1 P1 | 10.37596911 |
|  | PC | PC(35:6)+H | 764.5224835 | C43 H75 O8 N1 P1 | 7.804545228 |
|  | PC | PC(35:4)+H | 768.5537835 | C43 H79 O8 N1 P1 | 9.45768104 |
|  | PC | PC(35:3)+H | 770.5694335 | C43 H81 O8 N1 P1 | 9.601077208 |
|  | PC | PC(35:3)+H | 770.5694335 | C43 H81 O8 N1 P1 | 10.37645467 |
|  | PC | PC(35:3)+H | 770.5694335 | C43 H81 O8 N1 P1 | 9.875265548 |
|  | PC | PC(36:6)+H | 778.5381335 | C44 H77 O8 N1 P1 | 8.021271542 |
|  | PC | PC(36:5)+H | 780.5537835 | C44 H79 O8 N1 P1 | 8.855308325 |
|  | PC | PC(36:5)+H | 780.5537835 | C44 H79 O8 N1 P1 | 9.130339823 |
|  | PC | PC(36:4)+H | 782.5694335 | C44 H81 O8 N1 P1 | 9.721635192 |
|  | PC | PC(36:3)+H | 784.5850835 | C44 H83 O8 N1 P1 | 10.88260671 |
|  | PC | PC(36:3)+H | 784.5850835 | C44 H83 O8 N1 P1 | 10.51692099 |
|  | PC | PC(37:5)+H | 794.5694335 | C45 H81 O8 N1 P1 | 9.648 |
|  | PC | PC(37:4)+H | 796.5850835 | C45 H83 O8 N1 P1 | 10.42946455 |
|  | PC | PC(37:3)+H | 798.6007335 | C45 H85 O8 N1 P1 | 11.16315673 |
|  | PC | PC(38:3e)+H | 798.6371185 | C46 H89 O7 N1 P1 | 13.29552562 |
|  | PC | PC(38:2p)+H | 798.6371185 | C46 H89 O7 N1 P1 | 12.296 |
|  | PC | PC(38:6)+H | 806.5694335 | C46 H81 O8 N1 P1 | 9.264368965 |
|  | PC | PC(38:4)+H | 810.6007335 | C46 H85 O8 N1 P1 | 10.87270226 |
|  | PC | PC(38:3)+H | 812.6163835 | C46 H87 O8 N1 P1 | 11.76454068 |
|  | PC | PC(40:2p)+H | 826.6684185 | C48 H93 O7 N1 P1 | 10.806 |
|  | PC | PC(32:0)+HCOO | 778.5603605 | C41 H81 O10 N1 P1 | 11.07230446 |
|  | PC | PC(36:1)+HCOO | 832.6073105 | C45 H87 O10 N1 P1 | 12.51049898 |
|  | PC | PC(34:1)+HCOO | 804.5760105 | C43 H83 O10 N1 P1 | 11.59186215 |
|  | PC | PC(34:1)+H | 760.5850835 | C42 H83 O8 N1 P1 | 11.15101041 |
|  | PC | PC(36:2)+H | 786.6007335 | C44 H85 O8 N1 P1 | 11.26849911 |
|  | PC | PC(36:1)+H | 788.6163835 | C44 H87 O8 N1 P1 | 12.1023 |
|  | PC | PC(33:4)+H | 740.5224835 | C41 H75 O8 N1 P1 | 9.976761332 |
|  | PC | PC(36:6)+H | 778.5381335 | C44 H77 O8 N1 P1 | 8.295326675 |
| Phosphatidylethanolamine | PE | PE(34:3)+H | 714.5068335 | C39 H73 O8 N1 P1 | 10.7 |
|  | PE | PE(34:3)+H | 714.5068335 | C39 H73 O8 N1 P1 | 10.2469349 |
|  | PE | PE(34:2)+H | 716.5224835 | C39 H75 O8 N1 P1 | 11.29258268 |
|  | PE | PE(35:3)+H | 728.5224835 | C40 H75 O8 N1 P1 | 9.521 |
|  | PE | PE(36:5)+H | 738.5068335 | C41 H73 O8 N1 P1 | 9.137843827 |
|  | PE | PE(55:3)+H | 1008.835483 | C60 H115 O8 N1 P1 | 17.63628132 |
|  | PE | PE(40:1)+H | 802.6320335 | C45 H89 O8 N1 P1 | 12.73879665 |
|  | PE | PE(41:3)+H | 812.6163835 | C46 H87 O8 N1 P1 | 11.11752119 |
|  | PE | PE(34:1)-H | 716.5235805 | C39 H75 O8 N1 P1 | 11.4155854 |
|  | PE | PE(34:2)-H | 714.5079305 | C39 H73 O8 N1 P1 | 11.01143709 |
|  | PE | PE(36:4)-H | 738.5079305 | C41 H73 O8 N1 P1 | 7.778662227 |
|  | PE | PE(36:3)-H | 740.5235805 | C41 H75 O8 N1 P1 | 8.593158005 |
|  | PE | PE(36:2)-H | 742.5392305 | C41 H77 O8 N1 P1 | 11.52214616 |
|  | PE | PE(36:2)-H | 742.5392305 | C41 H77 O8 N1 P1 | 9.414381022 |
|  | PE | PE(42:5)-H | 820.5861805 | C47 H83 O8 N1 P1 | 12.786 |
|  | PE | PE(36:3)+H | 742.5381335 | C41 H77 O8 N1 P1 | 10.74741104 |
|  | PE | PE(38:5)+H | 766.5381335 | C43 H77 O8 N1 P1 | 8.645 |
|  | PE | PE(47:4)+H | 894.6946335 | C52 H97 O8 N1 P1 | 14.317 |
|  | PE | PE(55:2)+H | 1010.851133 | C60 H117 O8 N1 P1 | 16.00012577 |
|  | PE | PE(55:1)+H | 1012.866783 | C60 H119 O8 N1 P1 | 19.5550634 |
|  | PE | PE(57:1)+H | 1040.898083 | C62 H123 O8 N1 P1 | 20.70338463 |
|  | PE | PE(34:3)-H | 712.4922805 | C39 H71 O8 N1 P1 | 9.921530833 |
|  | PE | PE(36:4)-H | 738.5079305 | C41 H73 O8 N1 P1 | 9.957584247 |
|  | PE | PE(36:3)-H | 740.5235805 | C41 H75 O8 N1 P1 | 11.00431855 |
|  | PE | PE(36:2)+H | 744.5537835 | C41 H79 O8 N1 P1 | 9.885 |
|  | PE | PE(34:2)+H | 716.5224835 | C39 H75 O8 N1 P1 | 10.66582881 |
|  | PE | PE(36:1)-H | 744.5548805 | C41 H79 O8 N1 P1 | 12.34690573 |
|  | PE | PE(41:3)-H | 810.6018305 | C46 H85 O8 N1 P1 | 13.38053014 |
| Phosphatidylglycerol | PG | PG(39:1)+NH4 | 836.6375135 | C45 H91 O10 N1 P1 | 11.469 |
|  | PG | PG(44:1)+NH4 | 906.7157635 | C50 H101 O10 N1 P1 | 12.323 |
|  | PG | PG(44:1)+NH4 | 906.7157635 | C50 H101 O10 N1 P1 | 13.142 |
|  | PG | PG(46:1)+NH4 | 934.7470635 | C52 H105 O10 N1 P1 | 12.52427781 |
|  | PG | PG(31:4)-H | 699.4242615 | C37 H64 O10 N0 P1 | 6.630895762 |
|  | PG | PG(32:5)-H | 711.4242615 | C38 H64 O10 N0 P1 | 7.392350558 |
|  | PG | PG(33:2)-H | 731.4868615 | C39 H72 O10 N0 P1 | 9.430507568 |
|  | PG | PG(33:0)-H | 735.5181615 | C39 H76 O10 N0 P1 | 10.69870147 |
|  | PG | PG(35:1)-H | 761.5338115 | C41 H78 O10 N0 P1 | 11.30022179 |
|  | PG | PG(36:3)-H | 771.5181615 | C42 H76 O10 N0 P1 | 9.427788613 |
|  | PG | PG(33:1)-H | 733.5025115 | C39 H74 O10 N0 P1 | 10.34395169 |
|  | PG | PG(44:3)-H | 883.6433615 | C50 H92 O10 N0 P1 | 13.543 |
|  | PG | PG(36:4)+NH4 | 788.5436135 | C42 H79 O10 N1 P1 | 8.331218335 |
|  | PG | PG(30:2p)-H | 673.4449965 | C36 H66 O9 N0 P1 | 6.058 |
|  | PG | PG(32:0)-H | 721.5025115 | C38 H74 O10 N0 P1 | 10.2049228 |
|  | PG | PG(34:5)-H | 739.4555615 | C40 H68 O10 N0 P1 | 10.86000882 |
|  | PG | PG(34:4)-H | 741.4712115 | C40 H70 O10 N0 P1 | 8.337901845 |
|  | PG | PG(34:4)-H | 741.4712115 | C40 H70 O10 N0 P1 | 11.59900685 |
|  | PG | PG(34:3)-H | 743.4868615 | C40 H72 O10 N0 P1 | 8.742034755 |
|  | PG | PG(34:1)-H | 747.5181615 | C40 H76 O10 N0 P1 | 10.29165869 |
|  | PG | PG(36:3)+NH4 | 790.5592635 | C42 H81 O10 N1 P1 | 9.11279714 |
|  | PG | PG(32:1)-H | 719.4868615 | C38 H72 O10 N0 P1 | 9.849659993 |
|  | PG | PG(34:3)-H | 743.4868615 | C40 H72 O10 N0 P1 | 9.116715403 |
|  | PG | PG(34:2)-H | 745.5025115 | C40 H74 O10 N0 P1 | 9.936949483 |
|  | PG | PG(34:2)-H | 745.5025115 | C40 H74 O10 N0 P1 | 9.494395957 |
|  | PG | PG(34:1)-H | 747.5181615 | C40 H76 O10 N0 P1 | 10.83034115 |
|  | PG | PG(34:0)-H | 749.5338115 | C40 H78 O10 N0 P1 | 11.16287544 |
| Phosphatidylinositol | PI | PI(33:2p)-H | 803.5079915 | C42 H76 O12 N0 P1 | 7.397393345 |
|  | PI | PI(34:1)-H | 835.5342065 | C43 H80 O13 N0 P1 | 10.03256766 |
|  | PI | PI(36:4)-H | 857.5185565 | C45 H78 O13 N0 P1 | 8.561785395 |
|  | PI | PI(36:3)-H | 859.5342065 | C45 H80 O13 N0 P1 | 9.64413064 |
|  | PI | PI(36:2)-H | 861.5498565 | C45 H82 O13 N0 P1 | 10.14462825 |
|  | PI | PI(34:3)-H | 831.5029065 | C43 H76 O13 N0 P1 | 8.447722307 |
|  | PI | PI(34:2)-H | 833.5185565 | C43 H78 O13 N0 P1 | 9.22033688 |
|  | PI | PI(36:6)-H | 853.4872565 | C45 H74 O13 N0 P1 | 6.79316507 |
|  | PI | PI(36:5)-H | 855.5029065 | C45 H76 O13 N0 P1 | 7.657985612 |
|  | PI | PI(48:0)-H | 1033.768957 | C57 H110 O13 N0 P1 | 15.535 |
|  | PI | PI(50:3)-H | 1055.753307 | C59 H108 O13 N0 P1 | 14.295 |
|  | PI | PI(32:0)+NH4 | 828.5596585 | C41 H83 O13 N1 P1 | 9.918297303 |
|  | PI | PI(50:2)+NH4 | 1076.810058 | C59 H115 O13 N1 P1 | 19.14439627 |
|  | PI | PI(32:3p)-H | 787.4766915 | C41 H72 O12 N0 P1 | 9.853583213 |
|  | PI | PI(49:3)-H | 1041.737657 | C58 H106 O13 N0 P1 | 13.731 |
|  | PI | PI(51:4)-H | 1067.753307 | C60 H108 O13 N0 P1 | 13.59081019 |
| Phosphatidylserine | PS | PS(33:0)-H | 748.5134105 | C39 H75 O10 N1 P1 | 9.415817487 |
|  | PS | PS(40:8)-H | 830.4977605 | C46 H73 O10 N1 P1 | 7.850129687 |
|  | PS | PS(39:4)-H | 824.5447105 | C45 H79 O10 N1 P1 | 8.881951668 |
| Phosphatidylinositol | PIP | PIP(54:0)-H | 1197.82919 | C63 H123 O16 N0 P2 | 14.82545681 |
|  | PIP | PIP(56:3)-H | 1219.81354 | C65 H121 O16 N0 P2 | 13.41749966 |
|  | PIP | PIP(56:2)-H | 1221.82919 | C65 H123 O16 N0 P2 | 14.19532795 |
|  | PIP | PIP(56:0)-H | 1225.86049 | C65 H127 O16 N0 P2 | 16.107 |
| Cardiolipin | CL | CL(68:6)-H | 1395.933655 | C77 H137 O17 P2 | 16.7186539 |
|  | CL | CL(68:5)-H | 1397.949305 | C77 H139 O17 P2 | 17.51359318 |
|  | CL | CL(68:4)-H | 1399.964955 | C77 H141 O17 P2 | 18.37129572 |
|  | CL | CL(68:3)-H | 1401.980605 | C77 H143 O17 P2 | 19.20195737 |
|  | CL | CL(70:8)-H | 1419.933655 | C79 H137 O17 P2 | 15.90906544 |
|  | CL | CL(70:7)-H | 1421.949305 | C79 H139 O17 P2 | 16.70807253 |
|  | CL | CL(70:6)-H | 1423.964955 | C79 H141 O17 P2 | 17.53555142 |
|  | CL | CL(72:11)-H | 1441.918005 | C81 H135 O17 P2 | 14.45738386 |
|  | CL | CL(72:10)-H | 1443.933655 | C81 H137 O17 P2 | 15.17443733 |
|  | CL | CL(72:9)-H | 1445.949305 | C81 H139 O17 P2 | 15.92735711 |
|  | CL | CL(72:8)-H | 1447.964955 | C81 H141 O17 P2 | 16.72208258 |
|  | CL | CL(72:7)-H | 1449.980605 | C81 H143 O17 P2 | 17.5784151 |
|  | CL | CL(74:11)-H | 1469.949305 | C83 H139 O17 P2 | 16.74428885 |
|  | CL | CL(70:5)-H | 1425.980605 | C79 H143 O17 P2 | 18.41052477 |
| Lysophosphatidic acid | LPA | LPA(18:1)-H | 435.2517165 | C21 H40 O7 N0 P1 | 2.918162378 |
| Lysophosphatidylcholine | LPC | LPC(16:0)+HCOO | 540.3306955 | C25 H51 O9 N1 P1 | 2.697213727 |
|  | LPC | LPC(18:1)+H | 522.3554185 | C26 H53 O7 N1 P1 | 2.672125745 |
|  | LPC | LPC(19:1)+H | 536.3710685 | C27 H55 O7 N1 P1 | 3.174770293 |
|  | LPC | LPC(19:0)+H | 538.3867185 | C27 H57 O7 N1 P1 | 4.582109978 |
|  | LPC | LPC(18:3)+H | 518.3241185 | C26 H49 O7 N1 P1 | 1.910842688 |
|  | LPC | LPC(18:2)+H | 520.3397685 | C26 H51 O7 N1 P1 | 2.187913413 |
|  | LPC | LPC(18:0)+H | 524.3710685 | C26 H55 O7 N1 P1 | 3.787631783 |
| Lysophosphatidylethanolamine | LPE | LPE(16:0)-H | 452.2782655 | C21 H43 O7 N1 P1 | 2.833913762 |
|  | LPE | LPE(18:3)-H | 474.2626155 | C23 H41 O7 N1 P1 | 2.002597122 |
|  | LPE | LPE(18:2)-H | 476.2782655 | C23 H43 O7 N1 P1 | 2.370723695 |
|  | LPE | LPE(18:0)-H | 480.3095655 | C23 H47 O7 N1 P1 | 4.006108568 |
| Lysophosphatidylglycerol | LPG | LPG(18:2)-H | 507.2728465 | C24 H44 O9 N0 P1 | 1.985148202 |
|  | LPG | LPG(16:1)-H | 481.2571965 | C22 H42 O9 N0 P1 | 2.025096917 |
| Monogalactosylmonoacylglycerol | MGMG | MGMG(16:0)+HCOO | 537.3280385 | C26 H49 O11 | 2.993506698 |
|  | MGMG | MGMG(18:3)+HCOO | 559.3123885 | C28 H47 O11 | 2.142018252 |
|  | MGMG | MGMG(18:2)+HCOO | 561.3280385 | C28 H49 O11 | 2.590140998 |
|  | MGMG | MGMG(18:1)+HCOO | 563.3436885 | C28 H51 O11 | 3.207906507 |
|  | MGMG | MGMG(18:0)+HCOO | 565.3593385 | C28 H53 O11 | 4.57548811 |
| Monogalactosyldiacylglycerol | MGDG | MGDG(32:2)+HCOO | 771.5264035 | C42 H75 O12 | 10.06781828 |
|  | MGDG | MGDG(32:1)+HCOO | 773.5420535 | C42 H77 O12 | 10.89249533 |
|  | MGDG | MGDG(32:0)+HCOO | 775.5577035 | C42 H79 O12 | 11.76167974 |
|  | MGDG | MGDG(33:3)+HCOO | 783.5264035 | C43 H75 O12 | 9.854346198 |
|  | MGDG | MGDG(33:1)+HCOO | 787.5577035 | C43 H79 O12 | 11.36192574 |
|  | MGDG | MGDG(34:7)+HCOO | 789.4794535 | C44 H69 O12 | 8.063248093 |
|  | MGDG | MGDG(33:0)+HCOO | 789.5733535 | C43 H81 O12 | 12.2362478 |
|  | MGDG | MGDG(34:6)+HCOO | 791.4951035 | C44 H71 O12 | 7.823052015 |
|  | MGDG | MGDG(34:5)+HCOO | 793.5107535 | C44 H73 O12 | 8.655903175 |
|  | MGDG | MGDG(34:4)+HCOO | 795.5264035 | C44 H75 O12 | 9.467586718 |
|  | MGDG | MGDG(34:3)+HCOO | 797.5420535 | C44 H77 O12 | 9.865629887 |
|  | MGDG | MGDG(34:3)+HCOO | 797.5420535 | C44 H77 O12 | 10.39164263 |
|  | MGDG | MGDG(34:2)+HCOO | 799.5577035 | C44 H79 O12 | 11.08291982 |
|  | MGDG | MGDG(34:1)+HCOO | 801.5733535 | C44 H81 O12 | 11.8208494 |
|  | MGDG | MGDG(34:0)+HCOO | 803.5890035 | C44 H83 O12 | 12.71056462 |
|  | MGDG | MGDG(35:6)+HCOO | 805.5107535 | C45 H73 O12 | 8.304456448 |
|  | MGDG | MGDG(35:5)+HCOO | 807.5264035 | C45 H75 O12 | 9.42509111 |
|  | MGDG | MGDG(35:4)+HCOO | 809.5420535 | C45 H77 O12 | 9.984111957 |
|  | MGDG | MGDG(35:3)+HCOO | 811.5577035 | C45 H79 O12 | 10.89751665 |
|  | MGDG | MGDG(36:9)+HCOO | 813.4794535 | C46 H69 O12 | 7.86847421 |
|  | MGDG | MGDG(35:2)+HCOO | 813.5733535 | C45 H81 O12 | 11.56273118 |
|  | MGDG | MGDG(36:7)+HCOO | 817.5107535 | C46 H73 O12 | 9.022101472 |
|  | MGDG | MGDG(36:6)+HCOO | 819.5264035 | C46 H75 O12 | 8.857275385 |
|  | MGDG | MGDG(36:6)+HCOO | 819.5264035 | C46 H75 O12 | 8.349560968 |
|  | MGDG | MGDG(36:5)+HCOO | 821.5420535 | C46 H77 O12 | 9.644590623 |
|  | MGDG | MGDG(36:4)+HCOO | 823.5577035 | C46 H79 O12 | 10.44258514 |
|  | MGDG | MGDG(36:3)+HCOO | 825.5733535 | C46 H81 O12 | 11.27733324 |
|  | MGDG | MGDG(36:2)+HCOO | 827.5890035 | C46 H83 O12 | 12.0078816 |
|  | MGDG | MGDG(38:9)+HCOO | 841.5107535 | C48 H73 O12 | 8.406064297 |
|  | MGDG | MGDG(38:8)+HCOO | 843.5264035 | C48 H75 O12 | 9.387860682 |
|  | MGDG | MGDG(38:6)+HCOO | 847.5577035 | C48 H79 O12 | 9.883661652 |
|  | MGDG | MGDG(38:4)+HCOO | 851.5890035 | C48 H83 O12 | 11.45774073 |
|  | MGDG | MGDG(32:4)+HCOO | 767.4951035 | C42 H71 O12 | 8.40588493 |
|  | MGDG | MGDG(32:3)+HCOO | 769.5107535 | C42 H73 O12 | 9.291830412 |
|  | MGDG | MGDG(38:7)+HCOO | 845.5420535 | C48 H77 O12 | 10.06221899 |
| Digalactosyldiacylglycerol | DGDG | DGDG(31:3)+HCOO | 917.5479285 | C47 H81 O17 | 10.14023669 |
|  | DGDG | DGDG(32:3)+HCOO | 931.5635785 | C48 H83 O17 | 8.678533938 |
|  | DGDG | DGDG(32:0)+HCOO | 937.6105285 | C48 H89 O17 | 11.03620834 |
|  | DGDG | DGDG(33:0)+HCOO | 951.6261785 | C49 H91 O17 | 11.49823284 |
|  | DGDG | DGDG(34:6)+HCOO | 953.5479285 | C50 H81 O17 | 6.918352363 |
|  | DGDG | DGDG(34:5)+HCOO | 955.5635785 | C50 H83 O17 | 7.794149715 |
|  | DGDG | DGDG(34:4)+HCOO | 957.5792285 | C50 H85 O17 | 8.646161263 |
|  | DGDG | DGDG(34:3)+HCOO | 959.5948785 | C50 H87 O17 | 9.618643057 |
|  | DGDG | DGDG(34:2)+HCOO | 961.6105285 | C50 H89 O17 | 10.33796428 |
|  | DGDG | DGDG(34:1)+HCOO | 963.6261785 | C50 H91 O17 | 11.10808979 |
|  | DGDG | DGDG(34:0)+HCOO | 965.6418285 | C50 H93 O17 | 11.9600764 |
|  | DGDG | DGDG(35:3)+HCOO | 973.6105285 | C51 H89 O17 | 10.1570556 |
|  | DGDG | DGDG(36:6)+HCOO | 981.5792285 | C52 H85 O17 | 8.024368638 |
|  | DGDG | DGDG(37:12)+HCOO | 983.5009785 | C53 H75 O17 | 9.967657993 |
|  | DGDG | DGDG(36:5)+HCOO | 983.5948785 | C52 H87 O17 | 8.84666941 |
|  | DGDG | DGDG(36:4)+HCOO | 985.6105285 | C52 H89 O17 | 9.694762177 |
|  | DGDG | DGDG(36:3)+HCOO | 987.6261785 | C52 H91 O17 | 10.66470222 |
|  | DGDG | DGDG(36:2)+HCOO | 989.6418285 | C52 H93 O17 | 11.31733975 |
|  | DGDG | DGDG(36:0)+HCOO | 993.6731285 | C52 H97 O17 | 12.88887188 |
|  | DGDG | DGDG(37:3)+HCOO | 1001.641829 | C53 H93 O17 | 11.14141952 |
|  | DGDG | DGDG(38:7)+HCOO | 1007.594879 | C54 H87 O17 | 8.447344352 |
|  | DGDG | DGDG(40:0)+HCOO | 1049.735729 | C56 H105 O17 | 15.626 |
|  | DGDG | DGDG(42:1)+HCOO | 1075.751379 | C58 H107 O17 | 14.74807269 |
|  | DGDG | DGDG(47:13)+HCOO | 1121.641829 | C63 H93 O17 | 9.061405557 |
|  | DGDG | DGDG(49:16)+HCOO | 1143.626179 | C65 H91 O17 | 7.521841808 |
|  | DGDG | DGDG(31:4)+HCOO | 915.5322785 | C47 H79 O17 | 7.99782068 |
|  | DGDG | DGDG(31:3)+HCOO | 917.5479285 | C47 H81 O17 | 9.569 |
|  | DGDG | DGDG(39:1)+HCOO | 1033.704429 | C55 H101 O17 | 14.31084497 |
| Sulphoquinovosyldiacylglycerol | SQDG | SQDG(20:6)+HCOO | 659.2379055 | C30 H43 O14 S1 | 2.258359528 |
|  | SQDG | SQDG(33:3)+HCOO | 847.4883055 | C43 H75 O14 S1 | 3.623194968 |
|  | SQDG | SQDG(33:1)+HCOO | 851.5196055 | C43 H79 O14 S1 | 4.022 |
|  | SQDG | SQDG(34:3)+HCOO | 861.5039555 | C44 H77 O14 S1 | 9.728810833 |
|  | SQDG | SQDG(36:6)+HCOO | 883.4883055 | C46 H75 O14 S1 | 8.26849216 |
|  | SQDG | SQDG(36:5)+HCOO | 885.5039555 | C46 H77 O14 S1 | 9.015 |
|  | SQDG | SQDG(36:4)+HCOO | 887.5196055 | C46 H79 O14 S1 | 9.824141712 |
|  | SQDG | SQDG(38:9)+HCOO | 905.4726555 | C48 H73 O14 S1 | 6.683723123 |
|  | SQDG | SQDG(38:7)+HCOO | 909.5039555 | C48 H77 O14 S1 | 8.403962293 |
|  | SQDG | SQDG(38:6)+HCOO | 911.5196055 | C48 H79 O14 S1 | 9.371900458 |
|  | SQDG | SQDG(40:8)+HCOO | 935.5196055 | C50 H79 O14 S1 | 11.21724594 |
|  | SQDG | SQDG(40:8)+HCOO | 935.5196055 | C50 H79 O14 S1 | 12.25669578 |
|  | SQDG | SQDG(40:7)+HCOO | 937.5352555 | C50 H81 O14 S1 | 12.35993535 |
|  | SQDG | SQDG(40:7)+HCOO | 937.5352555 | C50 H81 O14 S1 | 11.89697193 |
|  | SQDG | SQDG(44:8)+HCOO | 991.5822055 | C54 H87 O14 S1 | 6.453844462 |
|  | SQDG | SQDG(41:8)+HCOO | 949.5352555 | C51 H81 O14 S1 | 10.47 |
| Diacylglycerol | DAG | DG(32:1)+NH4 | 584.5248505 | C35 H70 O5 N1 | 12.6328288 |
|  | DAG | DG(32:3)+Na | 585.4489465 | C35 H62 O5 Na1 | 7.816384252 |
|  | DAG | DG(32:3)+Na | 585.4489465 | C35 H62 O5 Na1 | 8.304541547 |
|  | DAG | DG(32:3)+Na | 585.4489465 | C35 H62 O5 Na1 | 6.922119877 |
|  | DAG | DG(32:0)+NH4 | 586.5405005 | C35 H72 O5 N1 | 13.09540928 |
|  | DAG | DG(33:6)+NH4 | 588.4622505 | C36 H62 O5 N1 | 7.944 |
|  | DAG | DG(32:1)+Na | 589.4802465 | C35 H66 O5 Na1 | 8.729 |
|  | DAG | DG(33:3)+NH4 | 594.5092005 | C36 H68 O5 N1 | 11.13949277 |
|  | DAG | DG(34:4p)+Na | 595.4696815 | C37 H64 O4 Na1 | 17.74521467 |
|  | DAG | DG(34:4p)+Na | 595.4696815 | C37 H64 O4 Na1 | 8.86728176 |
|  | DAG | DG(34:3p)+Na | 597.4853315 | C37 H66 O4 Na1 | 9.65616455 |
|  | DAG | DG(34:2p)+Na | 599.5009815 | C37 H68 O4 Na1 | 11.677 |
|  | DAG | DG(34:4)+NH4 | 606.5092005 | C37 H68 O5 N1 | 11.19377238 |
|  | DAG | DG(34:4)+NH4 | 606.5092005 | C37 H68 O5 N1 | 10.7670008 |
|  | DAG | DG(34:1)+NH4 | 612.5561505 | C37 H74 O5 N1 | 13.14108876 |
|  | DAG | DG(35:4)+NH4 | 620.5248505 | C38 H70 O5 N1 | 11.2127751 |
|  | DAG | DG(35:3)+NH4 | 622.5405005 | C38 H72 O5 N1 | 12.10923325 |
|  | DAG | DG(35:3)+NH4 | 622.5405005 | C38 H72 O5 N1 | 10.16508614 |
|  | DAG | DG(35:2)+NH4 | 624.5561505 | C38 H74 O5 N1 | 12.82542582 |
|  | DAG | DG(36:6)+NH4 | 630.5092005 | C39 H68 O5 N1 | 8.856249163 |
|  | DAG | DG(36:5)+NH4 | 632.5248505 | C39 H70 O5 N1 | 9.650773455 |
|  | DAG | DG(36:4)+NH4 | 634.5405005 | C39 H72 O5 N1 | 11.6807572 |
|  | DAG | DG(36:3)+NH4 | 636.5561505 | C39 H74 O5 N1 | 12.38762895 |
|  | DAG | DG(36:2)+NH4 | 638.5718005 | C39 H76 O5 N1 | 13.21973974 |
|  | DAG | DG(36:1)+NH4 | 640.5874505 | C39 H78 O5 N1 | 14.24876306 |
|  | DAG | DG(36:1)+Na | 645.5428465 | C39 H74 O5 Na1 | 6.652 |
|  | DAG | DG(37:4)+NH4 | 648.5561505 | C40 H74 O5 N1 | 12.16246192 |
|  | DAG | DG(38:5)+NH4 | 660.5561505 | C41 H74 O5 N1 | 10.94 |
|  | DAG | DG(38:2)+NH4 | 666.6031005 | C41 H80 O5 N1 | 14.46806981 |
|  | DAG | DG(38:1)+NH4 | 668.6187505 | C41 H82 O5 N1 | 15.4179226 |
|  | DAG | DG(42:3)+NH4 | 720.6500505 | C45 H86 O5 N1 | 16.03948109 |
|  | DAG | DG(44:4)+NH4 | 746.6657005 | C47 H88 O5 N1 | 16.38488193 |
|  | DAG | DG(44:3)+NH4 | 748.6813505 | C47 H90 O5 N1 | 17.30668985 |
|  | DAG | DG(44:2)+NH4 | 750.6970005 | C47 H92 O5 N1 | 18.3512785 |
|  | DAG | DG(32:1)+Na | 589.4802465 | C35 H66 O5 Na1 | 7.419264417 |
|  | DAG | DG(34:4)+NH4 | 606.5092005 | C37 H68 O5 N1 | 8.7303228 |
|  | DAG | DG(34:3)+NH4 | 608.5248505 | C37 H70 O5 N1 | 10.39628206 |
|  | DAG | DG(34:3)+NH4 | 608.5248505 | C37 H70 O5 N1 | 9.622200107 |
|  | DAG | DG(34:2)+NH4 | 610.5405005 | C37 H72 O5 N1 | 12.31672469 |
|  | DAG | DG(34:2)+NH4 | 610.5405005 | C37 H72 O5 N1 | 11.09192297 |
|  | DAG | DG(32:1p)+Na | 573.4853315 | C35 H66 O4 Na1 | 9.142 |
|  | DAG | DG(32:1p)+Na | 573.4853315 | C35 H66 O4 Na1 | 8.429 |
|  | DAG | DG(32:1p)+Na | 573.4853315 | C35 H66 O4 Na1 | 11.61537316 |
|  | DAG | DG(34:4p)+Na | 595.4696815 | C37 H64 O4 Na1 | 10.15058574 |
|  | DAG | DG(34:3p)+Na | 597.4853315 | C37 H66 O4 Na1 | 10.91575058 |
|  | DAG | DG(33:3)+Na | 599.4645965 | C36 H64 O5 Na1 | 8.292140178 |
|  | DAG | DG(34:3)+NH4 | 608.5248505 | C37 H70 O5 N1 | 11.62425035 |
|  | DAG | DG(34:3)+NH4 | 608.5248505 | C37 H70 O5 N1 | 11.88977684 |
|  | DAG | DG(34:3)+NH4 | 608.5248505 | C37 H70 O5 N1 | 9.228932472 |
|  | DAG | DG(34:2)+NH4 | 610.5405005 | C37 H72 O5 N1 | 12.67711671 |
|  | DAG | DG(34:1)+NH4 | 612.5561505 | C37 H74 O5 N1 | 11.106 |
|  | DAG | DG(36:6)+NH4 | 630.5092005 | C39 H68 O5 N1 | 10.17515699 |
|  | DAG | DG(36:6)+NH4 | 630.5092005 | C39 H68 O5 N1 | 8.028211975 |
|  | DAG | DG(36:5)+NH4 | 632.5248505 | C39 H70 O5 N1 | 10.91335522 |
|  | DAG | DG(36:4)+NH4 | 634.5405005 | C39 H72 O5 N1 | 11.96934544 |
|  | DAG | DG(36:6)+Na | 635.4645965 | C39 H64 O5 Na1 | 10.17515699 |
|  | DAG | DG(36:3)+NH4 | 636.5561505 | C39 H74 O5 N1 | 12.64637995 |
|  | DAG | DG(37:3)+NH4 | 650.5718005 | C40 H76 O5 N1 | 13.1044306 |
|  | DAG | DG(38:7)+NH4 | 656.5248505 | C41 H70 O5 N1 | 10.53914172 |
|  | DAG | DG(38:6)+NH4 | 658.5405005 | C41 H72 O5 N1 | 11.23079437 |
|  | DAG | DG(38:5)+NH4 | 660.5561505 | C41 H74 O5 N1 | 11.98418639 |
|  | DAG | DG(38:4)+NH4 | 662.5718005 | C41 H76 O5 N1 | 12.69595686 |
|  | DAG | DG(38:3)+NH4 | 664.5874505 | C41 H78 O5 N1 | 13.68039504 |
|  | DAG | DG(40:6)+NH4 | 686.5718005 | C43 H76 O5 N1 | 12.20843626 |
|  | DAG | DG(40:6)+NH4 | 686.5718005 | C43 H76 O5 N1 | 11.2408334 |
|  | DAG | DG(40:3)+NH4 | 692.6187505 | C43 H82 O5 N1 | 14.82747608 |
|  | DAG | DG(41:3)+NH4 | 706.6344005 | C44 H84 O5 N1 | 15.41741753 |
|  | DAG | DG(36:4)+NH4 | 634.5405005 | C39 H72 O5 N1 | 9.736784767 |
|  | DAG | DG(42:4)+NH4 | 718.6344005 | C45 H84 O5 N1 | 15.16669851 |
|  | DAG | DG(42:2)+NH4 | 722.6657005 | C45 H88 O5 N1 | 16.90791763 |
| Triacylglycerol | TAG | TG(49:2)+NH4 | 834.7545155 | C52 H100 O6 N1 | 20.47988803 |
|  | TAG | TG(50:6)+NH4 | 840.7075655 | C53 H94 O6 N1 | 16.52781772 |
|  | TAG | TG(50:5)+NH4 | 842.7232155 | C53 H96 O6 N1 | 17.50731213 |
|  | TAG | TG(51:7)+NH4 | 852.7075655 | C54 H94 O6 N1 | 16.25679922 |
|  | TAG | TG(51:6)+NH4 | 854.7232155 | C54 H96 O6 N1 | 17.17524015 |
|  | TAG | TG(51:5)+NH4 | 856.7388655 | C54 H98 O6 N1 | 18.11663055 |
|  | TAG | TG(51:4)+NH4 | 858.7545155 | C54 H100 O6 N1 | 19.16949495 |
|  | TAG | TG(51:3)+NH4 | 860.7701655 | C54 H102 O6 N1 | 20.12096755 |
|  | TAG | TG(51:2)+NH4 | 862.7858155 | C54 H104 O6 N1 | 21.23964512 |
|  | TAG | TG(52:7)+NH4 | 866.7232155 | C55 H96 O6 N1 | 16.69383467 |
|  | TAG | TG(52:5)+NH4 | 870.7545155 | C55 H100 O6 N1 | 18.71995983 |
|  | TAG | TG(53:7)+NH4 | 880.7388655 | C56 H98 O6 N1 | 17.18808012 |
|  | TAG | TG(53:6)+NH4 | 882.7545155 | C56 H100 O6 N1 | 18.41642082 |
|  | TAG | TG(53:6)+NH4 | 882.7545155 | C56 H100 O6 N1 | 18.1359196 |
|  | TAG | TG(53:5)+NH4 | 884.7701655 | C56 H102 O6 N1 | 19.33023245 |
|  | TAG | TG(53:4)+NH4 | 886.7858155 | C56 H104 O6 N1 | 20.38600882 |
|  | TAG | TG(53:4)+NH4 | 886.7858155 | C56 H104 O6 N1 | 19.1751458 |
|  | TAG | TG(54:9)+NH4 | 890.7232155 | C57 H96 O6 N1 | 15.84154587 |
|  | TAG | TG(54:8)+NH4 | 892.7388655 | C57 H98 O6 N1 | 17.0286534 |
|  | TAG | TG(54:8)+NH4 | 892.7388655 | C57 H98 O6 N1 | 16.74379058 |
|  | TAG | TG(54:7)+NH4 | 894.7545155 | C57 H100 O6 N1 | 17.7860182 |
|  | TAG | TG(54:6)+NH4 | 896.7701655 | C57 H102 O6 N1 | 18.74697853 |
|  | TAG | TG(54:4)+NH4 | 900.8014655 | C57 H106 O6 N1 | 30.966 |
|  | TAG | TG(54:2)+NH4 | 904.8327655 | C57 H110 O6 N1 | 21.68660272 |
|  | TAG | TG(55:7)+NH4 | 908.7701655 | C58 H102 O6 N1 | 18.40310848 |
|  | TAG | TG(55:6)+NH4 | 910.7858155 | C58 H104 O6 N1 | 19.64427923 |
|  | TAG | TG(55:5)+NH4 | 912.8014655 | C58 H106 O6 N1 | 20.55098803 |
|  | TAG | TG(55:4)+NH4 | 914.8171155 | C58 H108 O6 N1 | 20.40672113 |
|  | TAG | TG(56:10)+NH4 | 916.7388655 | C59 H98 O6 N1 | 15.85442065 |
|  | TAG | TG(56:9)+NH4 | 918.7545155 | C59 H100 O6 N1 | 15.82591341 |
|  | TAG | TG(56:9)+NH4 | 918.7545155 | C59 H100 O6 N1 | 16.74510652 |
|  | TAG | TG(56:8)+NH4 | 920.7701655 | C59 H102 O6 N1 | 18.09027338 |
|  | TAG | TG(56:8)+NH4 | 920.7701655 | C59 H102 O6 N1 | 16.76131818 |
|  | TAG | TG(56:6)+NH4 | 924.8014655 | C59 H106 O6 N1 | 18.75326445 |
|  | TAG | TG(57:5)+NH4 | 940.8327655 | C60 H110 O6 N1 | 20.55474048 |
|  | TAG | TG(57:4)+NH4 | 942.8484155 | C60 H112 O6 N1 | 21.49 |
|  | TAG | TG(58:9)+NH4 | 946.7858155 | C61 H104 O6 N1 | 15.86254427 |
|  | TAG | TG(58:6)+NH4 | 952.8327655 | C61 H110 O6 N1 | 20.94840828 |
|  | TAG | TG(62:13)+NH4 | 994.7858155 | C65 H104 O6 N1 | 15.95939286 |
|  | TAG | TG(65:4)+NH4 | 1054.973615 | C68 H128 O6 N1 | 23.42184795 |
|  | TAG | TG(29:3)+NH4 | 552.4258655 | C32 H58 O6 N1 | 3.890543275 |
|  | TAG | TG(31:3)+NH4 | 580.4571655 | C34 H62 O6 N1 | 6.416522917 |
|  | TAG | TG(31:2)+NH4 | 582.4728155 | C34 H64 O6 N1 | 6.948845935 |
|  | TAG | TG(33:5)+NH4 | 604.4571655 | C36 H62 O6 N1 | 4.88775685 |
|  | TAG | TG(34:4p)+NH4 | 604.4935505 | C37 H66 O5 N1 | 9.988 |
|  | TAG | TG(33:4)+NH4 | 606.4728155 | C36 H64 O6 N1 | 6.010408288 |
|  | TAG | TG(34:3)+NH4 | 622.5041155 | C37 H68 O6 N1 | 8.813353688 |
|  | TAG | TG(34:2)+NH4 | 624.5197655 | C37 H70 O6 N1 | 7.49294205 |
|  | TAG | TG(36:6e)+NH4 | 630.5092005 | C39 H68 O5 N1 | 9.213 |
|  | TAG | TG(36:5)+NH4 | 646.5041155 | C39 H68 O6 N1 | 7.96252221 |
|  | TAG | TG(36:3)+NH4 | 650.5354155 | C39 H72 O6 N1 | 8.785416882 |
|  | TAG | TG(36:2)+NH4 | 652.5510655 | C39 H74 O6 N1 | 8.713526653 |
|  | TAG | TG(36:2)+NH4 | 652.5510655 | C39 H74 O6 N1 | 9.380851433 |
|  | TAG | TG(36:1)+NH4 | 654.5667155 | C39 H76 O6 N1 | 9.223 |
|  | TAG | TG(38:5p)+NH4 | 658.5405005 | C41 H72 O5 N1 | 10.18 |
|  | TAG | TG(37:2)+NH4 | 666.5667155 | C40 H76 O6 N1 | 9.305293872 |
|  | TAG | TG(38:6)+NH4 | 672.5197655 | C41 H70 O6 N1 | 7.139214498 |
|  | TAG | TG(38:5)+NH4 | 674.5354155 | C41 H72 O6 N1 | 7.0124025 |
|  | TAG | TG(38:3)+NH4 | 678.5667155 | C41 H76 O6 N1 | 8.762718677 |
|  | TAG | TG(46:0e)+NH4 | 782.7596005 | C49 H100 O5 N1 | 15.602 |
|  | TAG | TG(47:0)+NH4 | 810.7545155 | C50 H100 O6 N1 | 15.6891533 |
|  | TAG | TG(48:2)+NH4 | 820.7388655 | C51 H98 O6 N1 | 19.53662233 |
|  | TAG | TG(38:4)+NH4 | 676.5510655 | C41 H74 O6 N1 | 7.928850693 |
|  | TAG | TG(38:2)+NH4 | 680.5823655 | C41 H78 O6 N1 | 9.863 |
|  | TAG | TG(56:7)+NH4 | 922.7858155 | C59 H104 O6 N1 | 17.78836648 |
|  | TAG | TG(56:5)+NH4 | 926.8171155 | C59 H108 O6 N1 | 19.94212198 |
|  | TAG | TG(30:4)+NH4 | 564.4258655 | C33 H58 O6 N1 | 4.80297606 |
|  | TAG | TG(34:6e)+NH4 | 602.4779005 | C37 H64 O5 N1 | 9.206417508 |
|  | TAG | TG(34:2)+NH4 | 624.5197655 | C37 H70 O6 N1 | 8.733675717 |
|  | TAG | TG(34:2)+NH4 | 624.5197655 | C37 H70 O6 N1 | 9.379400988 |
|  | TAG | TG(36:6)+NH4 | 644.4884655 | C39 H66 O6 N1 | 7.142079788 |
|  | TAG | TG(36:5)+NH4 | 646.5041155 | C39 H68 O6 N1 | 6.988527292 |
|  | TAG | TG(44:0)+NH4 | 768.7075655 | C47 H94 O6 N1 | 19.30195247 |
|  | TAG | TG(45:0)+NH4 | 782.7232155 | C48 H96 O6 N1 | 15.69374076 |
|  | TAG | TG(46:2)+NH4 | 792.7075655 | C49 H94 O6 N1 | 18.25968628 |
|  | TAG | TG(46:1)+NH4 | 794.7232155 | C49 H96 O6 N1 | 19.3249235 |
|  | TAG | TG(46:0)+NH4 | 796.7388655 | C49 H98 O6 N1 | 20.5215152 |
|  | TAG | TG(47:1)+NH4 | 808.7388655 | C50 H98 O6 N1 | 20.02849083 |
|  | TAG | TG(47:0)+NH4 | 810.7545155 | C50 H100 O6 N1 | 20.91549437 |
|  | TAG | TG(48:3)+NH4 | 818.7232155 | C51 H96 O6 N1 | 18.51536735 |
|  | TAG | TG(48:1)+NH4 | 822.7545155 | C51 H100 O6 N1 | 20.57266772 |
|  | TAG | TG(48:0)+NH4 | 824.7701655 | C51 H102 O6 N1 | 21.61721272 |
|  | TAG | TG(49:4)+NH4 | 830.7232155 | C52 H96 O6 N1 | 18.26938847 |
|  | TAG | TG(49:3)+NH4 | 832.7388655 | C52 H98 O6 N1 | 19.15304557 |
|  | TAG | TG(49:2)+NH4 | 834.7545155 | C52 H100 O6 N1 | 20.15018563 |
|  | TAG | TG(49:1)+NH4 | 836.7701655 | C52 H102 O6 N1 | 21.12119205 |
|  | TAG | TG(49:0)+NH4 | 838.7858155 | C52 H104 O6 N1 | 22.05867352 |
|  | TAG | TG(50:3)+NH4 | 846.7545155 | C53 H100 O6 N1 | 19.75768933 |
|  | TAG | TG(50:2)+NH4 | 848.7701655 | C53 H102 O6 N1 | 20.70425235 |
|  | TAG | TG(50:1)+NH4 | 850.7858155 | C53 H104 O6 N1 | 21.62177198 |
|  | TAG | TG(50:0)+NH4 | 852.8014655 | C53 H106 O6 N1 | 22.46315352 |
|  | TAG | TG(51:3)+NH4 | 860.7701655 | C54 H102 O6 N1 | 20.39871612 |
|  | TAG | TG(52:9)+NH4 | 862.6919155 | C55 H92 O6 N1 | 14.8896413 |
|  | TAG | TG(52:8)+NH4 | 864.7075655 | C55 H94 O6 N1 | 15.73550027 |
|  | TAG | TG(51:1)+NH4 | 864.8014655 | C54 H106 O6 N1 | 22.06018575 |
|  | TAG | TG(52:7)+NH4 | 866.7232155 | C55 H96 O6 N1 | 17.14544185 |
|  | TAG | TG(51:0)+NH4 | 866.8171155 | C54 H108 O6 N1 | 22.81319008 |
|  | TAG | TG(52:6)+NH4 | 868.7388655 | C55 H98 O6 N1 | 17.75976988 |
|  | TAG | TG(52:6)+NH4 | 868.7388655 | C55 H98 O6 N1 | 18.09498432 |
|  | TAG | TG(52:4)+NH4 | 872.7701655 | C55 H102 O6 N1 | 19.77954607 |
|  | TAG | TG(52:3)+NH4 | 874.7858155 | C55 H104 O6 N1 | 20.98655557 |
|  | TAG | TG(52:3)+NH4 | 874.7858155 | C55 H104 O6 N1 | 20.71486255 |
|  | TAG | TG(52:2)+NH4 | 876.8014655 | C55 H106 O6 N1 | 21.64609683 |
|  | TAG | TG(53:8)+NH4 | 878.7232155 | C56 H96 O6 N1 | 16.18484589 |
|  | TAG | TG(52:1)+NH4 | 878.8171155 | C55 H108 O6 N1 | 22.4569408 |
|  | TAG | TG(52:0)+NH4 | 880.8327655 | C55 H110 O6 N1 | 23.13218885 |
|  | TAG | TG(54:10)+NH4 | 888.7075655 | C57 H94 O6 N1 | 15.22889739 |
|  | TAG | TG(53:3)+NH4 | 888.8014655 | C56 H106 O6 N1 | 21.53014078 |
|  | TAG | TG(53:3)+NH4 | 888.8014655 | C56 H106 O6 N1 | 21.2678839 |
|  | TAG | TG(53:2)+NH4 | 890.8171155 | C56 H108 O6 N1 | 22.13394893 |
|  | TAG | TG(53:1)+NH4 | 892.8327655 | C56 H110 O6 N1 | 22.80788062 |
|  | TAG | TG(54:6)+NH4 | 896.7701655 | C57 H102 O6 N1 | 19.0487169 |
|  | TAG | TG(54:5)+NH4 | 898.7858155 | C57 H104 O6 N1 | 19.84618568 |
|  | TAG | TG(54:4)+NH4 | 900.8014655 | C57 H106 O6 N1 | 19.76861828 |
|  | TAG | TG(54:4)+NH4 | 900.8014655 | C57 H106 O6 N1 | 20.80409792 |
|  | TAG | TG(54:3)+NH4 | 902.8171155 | C57 H108 O6 N1 | 21.73510912 |
|  | TAG | TG(54:2)+NH4 | 904.8327655 | C57 H110 O6 N1 | 22.49862308 |
|  | TAG | TG(54:1)+NH4 | 906.8484155 | C57 H112 O6 N1 | 23.125251 |
|  | TAG | TG(54:0)+NH4 | 908.8640655 | C57 H114 O6 N1 | 23.69456947 |
|  | TAG | TG(55:4)+NH4 | 914.8171155 | C58 H108 O6 N1 | 21.49385553 |
|  | TAG | TG(56:10)+NH4 | 916.7388655 | C59 H98 O6 N1 | 16.18857396 |
|  | TAG | TG(55:3)+NH4 | 916.8327655 | C58 H110 O6 N1 | 22.18651693 |
|  | TAG | TG(56:9)+NH4 | 918.7545155 | C59 H100 O6 N1 | 17.11285702 |
|  | TAG | TG(55:2)+NH4 | 918.8484155 | C58 H112 O6 N1 | 22.168584 |
|  | TAG | TG(55:2)+NH4 | 918.8484155 | C58 H112 O6 N1 | 22.86193282 |
|  | TAG | TG(55:1)+NH4 | 920.8640655 | C58 H114 O6 N1 | 23.41723713 |
|  | TAG | TG(56:7)+NH4 | 922.7858155 | C59 H104 O6 N1 | 19.10631293 |
|  | TAG | TG(56:6)+NH4 | 924.8014655 | C59 H106 O6 N1 | 20.24092633 |
|  | TAG | TG(56:6)+NH4 | 924.8014655 | C59 H106 O6 N1 | 19.06208537 |
|  | TAG | TG(56:5)+NH4 | 926.8171155 | C59 H108 O6 N1 | 20.28015543 |
|  | TAG | TG(56:5)+NH4 | 926.8171155 | C59 H108 O6 N1 | 21.10763367 |
|  | TAG | TG(56:4)+NH4 | 928.8327655 | C59 H110 O6 N1 | 21.03471615 |
|  | TAG | TG(56:4)+NH4 | 928.8327655 | C59 H110 O6 N1 | 21.9574242 |
|  | TAG | TG(56:4)+NH4 | 928.8327655 | C59 H110 O6 N1 | 20.75358543 |
|  | TAG | TG(56:3)+NH4 | 930.8484155 | C59 H112 O6 N1 | 22.555308 |
|  | TAG | TG(56:2)+NH4 | 932.8640655 | C59 H114 O6 N1 | 23.16114328 |
|  | TAG | TG(56:2)+NH4 | 932.8640655 | C59 H114 O6 N1 | 22.53588362 |
|  | TAG | TG(57:6)+NH4 | 938.8171155 | C60 H108 O6 N1 | 20.81789925 |
|  | TAG | TG(57:6)+NH4 | 938.8171155 | C60 H108 O6 N1 | 19.63095233 |
|  | TAG | TG(57:5)+NH4 | 940.8327655 | C60 H110 O6 N1 | 21.65661078 |
|  | TAG | TG(58:11)+NH4 | 942.7545155 | C61 H100 O6 N1 | 16.54267007 |
|  | TAG | TG(57:4)+NH4 | 942.8484155 | C60 H112 O6 N1 | 22.3581164 |
|  | TAG | TG(58:10)+NH4 | 944.7701655 | C61 H102 O6 N1 | 17.47679918 |
|  | TAG | TG(58:10)+NH4 | 944.7701655 | C61 H102 O6 N1 | 16.20335648 |
|  | TAG | TG(57:3)+NH4 | 944.8640655 | C60 H114 O6 N1 | 23.01540832 |
|  | TAG | TG(58:9)+NH4 | 946.7858155 | C61 H104 O6 N1 | 17.09921427 |
|  | TAG | TG(57:2)+NH4 | 946.8797155 | C60 H116 O6 N1 | 23.47763323 |
|  | TAG | TG(58:8)+NH4 | 948.8014655 | C61 H106 O6 N1 | 18.11423837 |
|  | TAG | TG(57:1)+NH4 | 948.8953655 | C60 H118 O6 N1 | 23.93186427 |
|  | TAG | TG(58:7)+NH4 | 950.8171155 | C61 H108 O6 N1 | 19.10122387 |
|  | TAG | TG(58:7)+NH4 | 950.8171155 | C61 H108 O6 N1 | 19.40600903 |
|  | TAG | TG(58:6)+NH4 | 952.8327655 | C61 H110 O6 N1 | 20.22505853 |
|  | TAG | TG(58:6)+NH4 | 952.8327655 | C61 H110 O6 N1 | 21.37769133 |
|  | TAG | TG(58:5)+NH4 | 954.8484155 | C61 H112 O6 N1 | 22.07409338 |
|  | TAG | TG(58:5)+NH4 | 954.8484155 | C61 H112 O6 N1 | 21.11921448 |
|  | TAG | TG(58:4)+NH4 | 956.8640655 | C61 H114 O6 N1 | 22.71650333 |
|  | TAG | TG(58:4)+NH4 | 956.8640655 | C61 H114 O6 N1 | 21.9519995 |
|  | TAG | TG(58:3)+NH4 | 958.8797155 | C61 H116 O6 N1 | 23.28787508 |
|  | TAG | TG(58:2)+NH4 | 960.8953655 | C61 H118 O6 N1 | 23.73301042 |
|  | TAG | TG(58:1)+NH4 | 962.9110155 | C61 H120 O6 N1 | 24.16432118 |
|  | TAG | TG(59:6)+NH4 | 966.8484155 | C62 H112 O6 N1 | 21.8461959 |
|  | TAG | TG(59:5)+NH4 | 968.8640655 | C62 H114 O6 N1 | 22.46020407 |
|  | TAG | TG(59:4)+NH4 | 970.8797155 | C62 H116 O6 N1 | 23.03107778 |
|  | TAG | TG(59:3)+NH4 | 972.8953655 | C62 H118 O6 N1 | 23.58131715 |
|  | TAG | TG(59:2)+NH4 | 974.9110155 | C62 H120 O6 N1 | 23.97148847 |
|  | TAG | TG(60:6)+NH4 | 980.8640655 | C63 H114 O6 N1 | 22.26411187 |
|  | TAG | TG(60:5)+NH4 | 982.8797155 | C63 H116 O6 N1 | 22.80537515 |
|  | TAG | TG(60:4)+NH4 | 984.8953655 | C63 H118 O6 N1 | 22.792 |
|  | TAG | TG(60:4)+NH4 | 984.8953655 | C63 H118 O6 N1 | 23.32995652 |
|  | TAG | TG(60:3)+NH4 | 986.9110155 | C63 H120 O6 N1 | 23.83794732 |
|  | TAG | TG(61:6)+NH4 | 994.8797155 | C64 H116 O6 N1 | 22.64187482 |
|  | TAG | TG(62:12)+NH4 | 996.8014655 | C65 H106 O6 N1 | 16.84813783 |
|  | TAG | TG(61:5)+NH4 | 996.8953655 | C64 H118 O6 N1 | 23.14371503 |
|  | TAG | TG(61:4)+NH4 | 998.9110155 | C64 H120 O6 N1 | 23.60796047 |
|  | TAG | TG(62:6)+NH4 | 1008.895365 | C65 H118 O6 N1 | 22.98460147 |
|  | TAG | TG(62:4)+NH4 | 1012.926665 | C65 H122 O6 N1 | 23.8680774 |
|  | TAG | TG(50:4)+NH4 | 844.7388655 | C53 H98 O6 N1 | 19.14041052 |
| Wax esters | WE | WE(21:1)+NH4 | 342.3366555 | H44 C21 O2 N1 | 2.845225027 |
|  | WE | WE(23:4)+NH4 | 364.3210055 | H42 C23 O2 N1 | 2.036273165 |
|  | WE | WE(23:1)+NH4 | 370.3679555 | H48 C23 O2 N1 | 4.043805303 |
|  | WE | WE(40:2)+NH4 | 606.6183555 | H80 C40 O2 N1 | 24.54010153 |
| Coenzyme | Co | Co(Q10)+NH4 | 880.7177355 | C59 H94 O4 N1 | 17.95960437 |
|  | Co | Co(Q9)+NH4 | 812.6551355 | C54 H86 O4 N1 | 16.08517128 |
| AcylGlcSitosterol ester | AGlcSiE | AGlcSiE(15:0)+NH4 | 818.6868305 | C50 H92 O7 N1 | 15.55118424 |
|  | AGlcSiE | AGlcSiE(16:0)+NH4 | 832.7024805 | C51 H94 O7 N1 | 15.95578438 |
|  | AGlcSiE | AGlcSiE(17:2)+NH4 | 842.6868305 | C52 H92 O7 N1 | 14.64392762 |
|  | AGlcSiE | AGlcSiE(17:1)+NH4 | 844.7024805 | C52 H94 O7 N1 | 15.61248641 |
|  | AGlcSiE | AGlcSiE(17:0)+NH4 | 846.7181305 | C52 H96 O7 N1 | 16.55062357 |
|  | AGlcSiE | AGlcSiE(18:3)+NH4 | 854.6868305 | C53 H92 O7 N1 | 14.20914676 |
|  | AGlcSiE | AGlcSiE(18:2)+NH4 | 856.7024805 | C53 H94 O7 N1 | 15.04462837 |
|  | AGlcSiE | AGlcSiE(18:1)+NH4 | 858.7181305 | C53 H96 O7 N1 | 16.02203092 |
|  | AGlcSiE | AGlcSiE(18:0)+NH4 | 860.7337805 | C53 H98 O7 N1 | 17.16388613 |
|  | AGlcSiE | AGlcSiE(20:4)+NH4 | 880.7024805 | C55 H94 O7 N1 | 14.2136003 |
|  | AGlcSiE | AGlcSiE(20:0)+NH4 | 888.7650805 | C55 H102 O7 N1 | 18.40442383 |
|  | AGlcSiE | AGlcSiE(23:0)+NH4 | 930.8120305 | C58 H108 O7 N1 | 20.24906757 |
|  | AGlcSiE | AGlcSiE(24:0)+NH4 | 944.8276805 | C59 H110 O7 N1 | 20.86750252 |
| Sitosterol ester | SiE | SiE(18:3)+NH4 | 692.6340055 | C47 H82 O2 N1 | 20.86786063 |
|  | SiE | SiE(18:2)+NH4 | 694.6496555 | C47 H84 O2 N1 | 21.81999407 |
|  | SiE | SiE(28:3)+NH4 | 832.7905055 | C57 H102 O2 N1 | 22.687 |
