# Supplementary material for: Effects of heat shock on photosynthesis-related characteristics and lipid profile of Cycas multipinnata and C. panzhihuaensis
Source: BMC Plant Biol. 2022 Sep 15;22:442. doi: 10.1186/s12870-022-03825-0 (PMC9476270; doi:10.1186/s12870-022-03825-0)
Supplement: Supplementary file 3 — Additional file 3. Changes in lipid molecular species of glycerophospholipids and lysophospholipids in Cycas multipinnata and C. panzhihuaensis subjected to heat stress. [file 12870_2022_3825_MOESM3_ESM.docx]

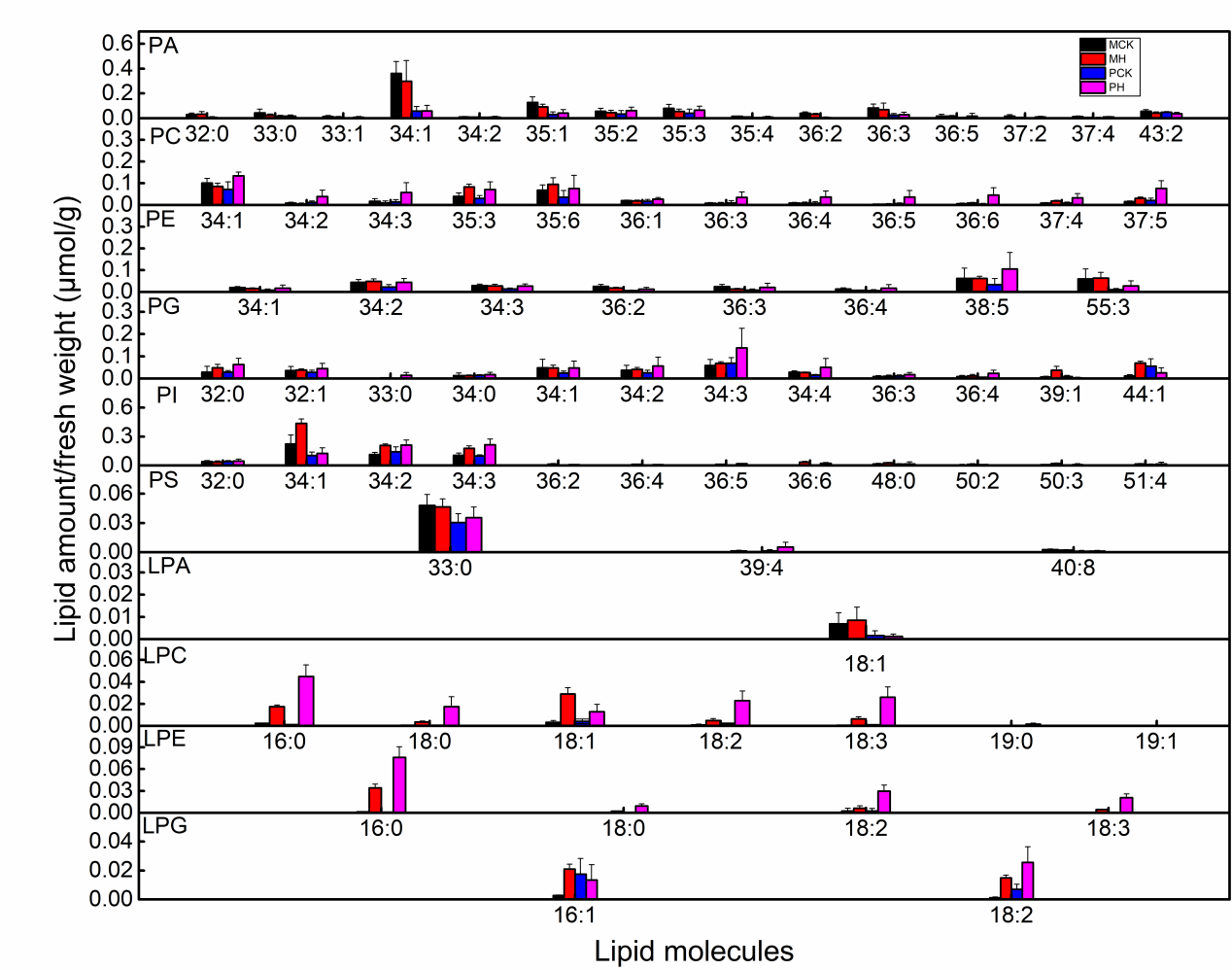


**Additional file 3.** Changes in lipid molecular species of glycerophospholipids and lysophospholipids in *Cycas multipinnata* and *C. panzhihuaensis* subjected to heat stress. MCK: *C. multipinnata* treated at control conditions; MH: *C. multipinnata* treated at heat stress; PCK: *C. panzhihuaensis* treated at control conditions; PH: *C. panzhihuaensis* treated at heat stress.
