# Supplementary material for: Effects of heat shock on photosynthesis-related characteristics and lipid profile of Cycas multipinnata and C. panzhihuaensis
Source: BMC Plant Biol. 2022 Sep 15;22:442. doi: 10.1186/s12870-022-03825-0 (PMC9476270; doi:10.1186/s12870-022-03825-0)
Supplement: Supplementary file 4 — Additional file 4. The digalactosyldiacylglycerol (DGDG)-monogalactosyldiacylglycerol (MGDG) ratio of Cycas multipinnata and C. panzhihuaensis subjected to heat stress. [file 12870_2022_3825_MOESM4_ESM.docx]

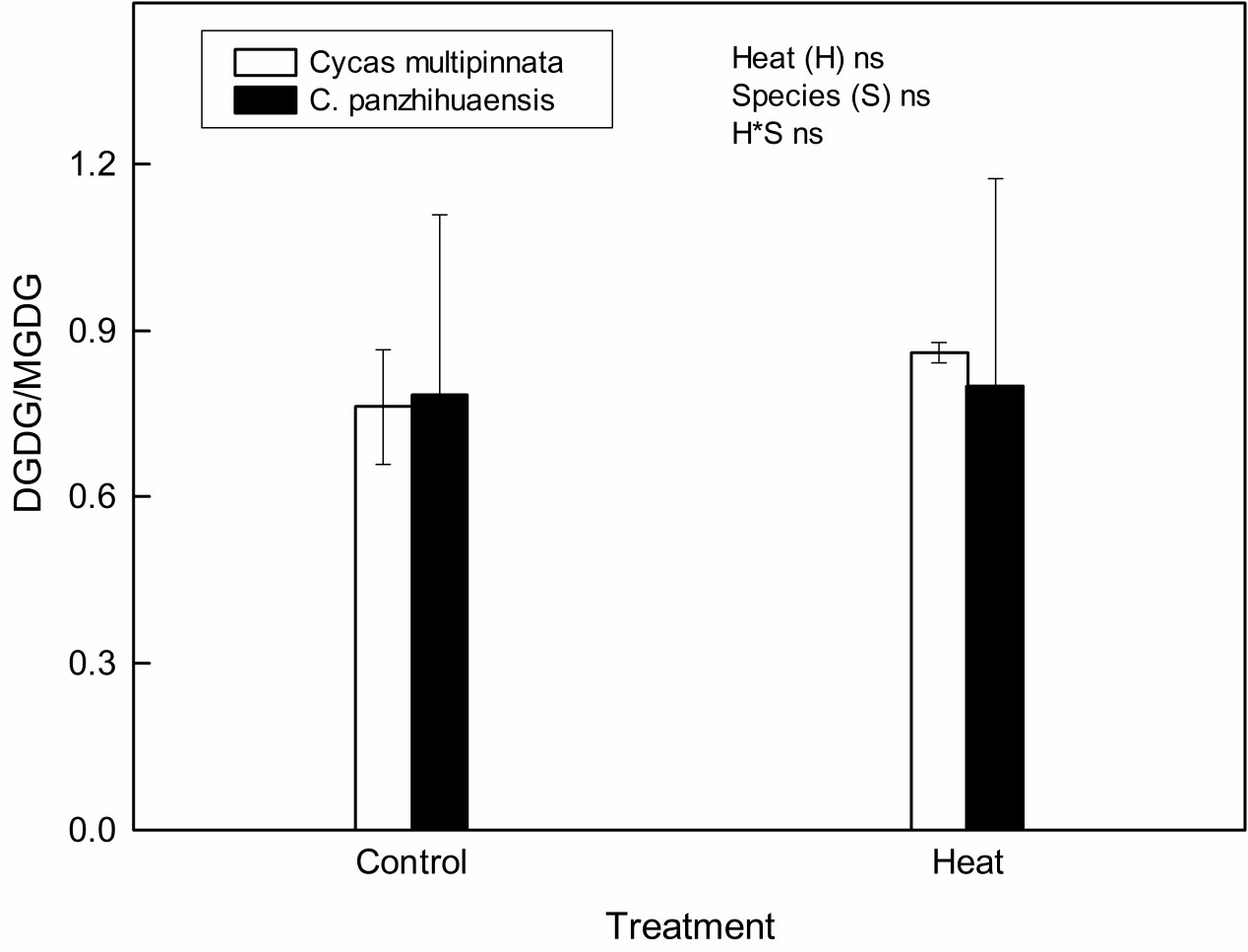


**Additional file 4.** The digalactosyldiacylglycerol (DGDG)-monogalactosyldiacylglycerol (MGDG) ratio of *Cycas multipinnata* and *C. panzhihuaensis* subjected to heat stress*.*
